# Exploring the abundance of oleate hydratases in the genus *Rhodococcus*—discovery of novel enzymes with complementary substrate scope

**DOI:** 10.1007/s00253-020-10627-7

**Published:** 2020-05-01

**Authors:** Hanna Busch, Fabio Tonin, Natália Alvarenga, Marcel van den Broek, Simona Lu, Jean-Marc Daran, Ulf Hanefeld, Peter-Leon Hagedoorn

**Affiliations:** Department of Biotechnology, Delft University of Technology, Van der Maasweg 9, 2629 HZ Delft, The Netherlands

**Keywords:** Fatty acid hydratase, Enzyme assay, Oleate hydratase, *Rhodococcus*, Water addition

## Abstract

Oleate hydratases (Ohys, EC 4.2.1.53) are a class of enzymes capable of selective water addition reactions to a broad range of unsaturated fatty acids leading to the respective chiral alcohols. Much research was dedicated to improving the applications of existing Ohys as well as to the identification of undescribed Ohys with potentially novel properties. This study focuses on the latter by exploring the genus *Rhodococcus* for its plenitude of oleate hydratases. Three different *Rhodococcus* clades showed the presence of oleate hydratases whereby each clade was represented by a specific oleate hydratase family (HFam). Phylogenetic and sequence analyses revealed HFam-specific patterns amongst conserved amino acids. Oleate hydratases from two *Rhodococcus* strains (HFam 2 and 3) were heterologously expressed in *Escherichia coli* and their substrate scope investigated. Here, both enzymes showed a complementary behaviour towards sterically demanding and multiple unsaturated fatty acids. Furthermore, this study includes the characterisation of the newly discovered *Rhodococcus pyridinivorans* Ohy. The steady-state kinetics of *R. pyridinivorans* Ohy was measured using a novel coupled assay based on the alcohol dehydrogenase and NAD^+^-dependent oxidation of 10-hydroxystearic acid.

**Electronic supplementary material:**

The online version of this article (10.1007/s00253-020-10627-7) contains supplementary material, which is available to authorized users.

## Introduction

*Rhodococcus* is a genus of aerobic, gram-positive bacteria and is known for its diverse biocatalytic activity towards a plethora of substrates (van der Geize and Dijkhuizen 2004; Jones and Goodfellow 2012; Kim et al. 2018; Busch et al. 2019). Aliphatic, aromatic or heterocyclic compounds as well as alicyclic hydrocarbons, cholesterol, nitriles and lignin have been shown to be converted by members of the versatile *Rhodococcus* family (van der Geize and Dijkhuizen 2004; Kim et al. 2018). The reason for their catabolic adaptability is explained by interacting factors such as their large genome sizes, the redundancy of biosynthetic pathways and the presence of large, linear plasmids often harbouring multiple copies of genes encoding degrading enzymes (van der Geize and Dijkhuizen 2004; Alvarez 2019; Zampolli et al. 2019).

The immense progress made in genomic studies enables a fast and extensive processing of bacterial genome information to designate gene functions to undescribed enzymes (Zampolli et al. 2019). This helps identifying novel biocatalysts in e.g. *Rhodococcus* and therefore increases the biotechnological potential for this catalytic powerhouse. One enzyme family that receives increasing attention is the class of hydratases (E.C. 4.2.1.x) which belong to the group of lyases. They catalyse the reversible water addition to π-bond systems and can be categorised in two groups based on their substrate scope: isolated double bonds or conjugated systems (Resch and Hanefeld 2015; Engleder and Pichler 2018).

**Scheme 1:**
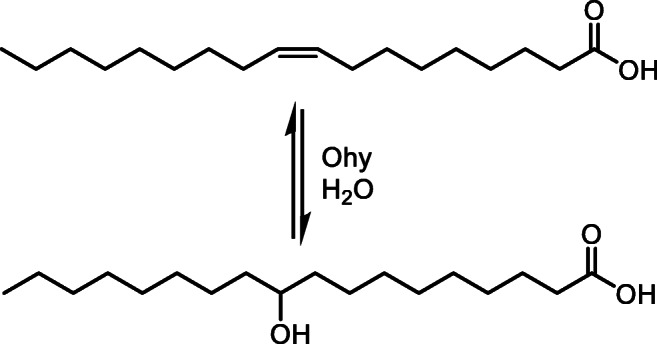
Oleate hydratase catalysed water addition to oleic acid (18:1, *cis*-9) yielding 10-hydroxystearic acid

Oleate hydratase (Ohy) belongs to the first group acting on isolated double bonds in fatty acids e.g. oleic acid to produce 10-hydroxystearic acid (Scheme 1) (Demming et al. 2018; Engleder and Pichler 2018; Serra et al. 2020). Up until now, all characterised Ohys are FAD-dependent. However, the precise role of FAD in the protein remains not fully understood (Engleder and Pichler 2018).

With the newly developed ‘Hydratase Engineering Database’, it is now possible to distinguish 11 homologous families (HFam) of fatty acid hydratases (Schmid et al. 2016). Sequence comparison of 2046 hydratase sequences exposed the presence of the 11 families (‘HFam 1 to 11’) whose members share an average sequence identity of 62%. With 1188 sequences, HFam 2 makes the largest group followed by HFam 1 and HFam 3 (Schmid et al. 2016).

Recently, the first Ohy from *Rhodococcus* was characterised and the structure elucidated (Lorenzen et al. 2017). The protein belongs to HFam 3 and thereby offers the first structural insight into a representative of this hydratase family. Unlike the other three crystallographically resolved Ohys which were all shown to be homodimers (Volkov et al. 2013; Engleder et al. 2015; Park et al. 2018), this protein has been described to be an active monomer in solution. The characterised Ohy from *R. erythropolis* CCM 2595, *Re*Ohy (CCM 2595), catalyses the conversion of a number of fatty acids exclusively in 10-position yielding the respective hydroxylated fatty acids (Lorenzen et al. 2017). This example again serves to show the rich abundance of diverse biocatalytic activities within *Rhodococcus* and demonstrates the possibilities of finding novel biocatalysts within this genus.

The aim of this study is to investigate the abundance of oleate hydratases within the genus *Rhodococcus*. An Orthologous Matrix (OMA) was established from multiple *Rhodococcus* strains whose data was made either publicly available or sequenced in-house. Through genome mining of those 43 *Rhodococcus* strains, 20 annotated oleate hydratases were revealed. Subsequently, the discovered oleate hydratases were analysed phylogenetically and categorised based on their HFam affiliation. Thereby, overall, three different groups of oleate hydratases have been distinguished (HFam 1 to 3) in *Rhodococcus*. One representative of each HFam 2 and HFam 3 was selected for heterologous expression, and an extensive whole-cell substrate screening was carried out to investigate differences in substrate acceptance. Additionally, the thereby newly discovered Ohy from *Rhodococcus pyridinivorans* DSM 20415 (HFam 2) was characterised and the properties were compared with the earlier described *Re*Ohy (CCM 2595) (HFam 3) (Lorenzen et al. 2017). As part of this characterisation, a novel enzyme assay for Ohys was developed.

## Material and methods

### Chemicals

All commercial chemicals were purchased from Sigma-Aldrich (Schnelldorf, Germany). The UltraClean Microbial DNA Isolation Kit was purchased from MOBIO Laboratories, Inc. (Uden, The Netherlands). *(R)*-10-hydroxystearic acid was obtained from InnoSyn (Geleen, The Netherlands; kind gift by Dr. Martin Schürmann).

### Bacterial strains and microorganisms

The *Rhodococcus pyridinivorans* DSM 20415 was purchased from the German Collection of Microorganisms and Cell Culture (Leibniz Institute DMSZ). Plasmid pBAD/His A and *Escherichia coli* TOP10 cells were obtained from Fisher Scientific (Landsmeer, The Netherlands). The GenBank accession numbers for the original and codon-optimised (including His-tag) nucleotide sequences of *Rp*Ohy are MN563120 and MN563121 respectively.

### Genomic DNA extraction

The genomic DNA of the strain *R. pyridinivorans* DSM 20415 was isolated from a cell pellet (50–100 mg) using the UltraClean Microbial DNA Isolation Kit following standard procedure.

### Whole-genome sequencing

Genomic DNA of *R. pyridinivorans* DSM 20415 was in-house sequenced on a MiSeq sequencer (Illumina, San Diego, CA) to obtain a 300 cycle paired-end library with an insert size of 550 bp using PCR-free library preparation, yielding 2.6 gigabases in total. De novo assembly was performed using SPAdes (version 3.9.0) (Bankevich et al. 2012). The assembled genome of *R. pyridinivorans* DSM 20415 were annotated by using Prokka (version 1.12) (Seemann 2014).

### Orthologous Matrix (OMA) and phylogenetic analysis

Pairwise orthologues of the proteome of the annotated genome were computed using the Orthologous Matrix (OMA) software (standalone version 2.2.0) (Train et al. 2017). A species tree was inferred based on the 1% most complete computed orthologous groups. MEGA (version 7.0.21) was used for visualization. Ohy protein sequences were aligned using MUSCLE (version 3.8.31) (Edgar 2004). Distance matrix was calculated by FastTree (version 2.1.9, JTT+CAT model) and then visualized by MEGA (Price et al. 2010).

### Cloning

The Ohy coding genes from *Rhodococcus erythropolis* PR4 (*Re*Ohy (PR4)) and from *Rhodococcus pyridinivorans* DSM 20415 (*Rp*Ohy) were used as templates for codon-optimised gene synthesis (BaseClear B.V., Leiden, NL). Both synthetic genes (*Re*Ohy (PR4) and *Rp*Ohy) were sub-cloned in pBAD/His A expression vectors and subsequently transformed into *E. coli* TOP10 cells following standard procedures.

### Protein expression and purification

*Re*Ohy (PR4) and *Rp*Ohy were expressed in *E. coli* TOP10 cells grown on LB medium. Pre-cultures were inoculated with a single colony and grown overnight at 37 °C with orbital shaking (180 rpm, Innova44, New Brunswick Scientific). The main cultures (1 l in a 5-l baffled shake flask) were inoculated with 5 ml of pre-culture and grown to an optical density of 0.6–0.8 at 37 °C with orbital shaking (180 rpm). The protein expression was induced by adding L-arabinose with a final concentration of 0.2%. After 16 h of growth at 25 °C, cells were harvested (15 min, 4 °C, 17,700 g), washed with 20 mM Tris-Cl (pH 8) and kept at 20 °C. Frozen cell pellets of *Rp*Ohy were thawed, resuspended in 20 mM Tris-Cl (pH 8) and disrupted by high pressure homogenisation (Constant Systems Ltd., UK). Subsequent centrifugation (15 min, 4 °C, 17,700 g) yielded cell debris and soluble protein fraction. The supernatant was applied on a Ni^2+^-NTA His-trap column (HisTrap FF, GE Healthcare, flow rate 1 ml/min) and purified on a NGC chromatography system (Bio-Rad Laboratories, Inc., US). The purified protein solution was desalted using a PD10 desalting column. The His-tag-purified *Rp*Ohy was afterwards separately applied on a HiLoad 26/60 Superdex 200 prep grade size-exclusion column (flow rate 2 ml/min). Respective fractions were collected and concentrated.

Oleate hydratase from *Elisabethkingia meningoseptica* (*Em-*OAH1) was expressed as a His-tagged protein using *E. coli* pBAD/His A–ohyA as previously described (Bevers et al. 2009). The enzyme was purified as described above for *Rp*Ohy.

### Fatty acid screening

For the fatty acid screening, *E. coli* TOP10 whole cells with overexpressed *Rp*Ohy, *Re*Ohy (PR4) or with an empty pBAD/His A expression vector were resuspended to a wet-cell content of 100 mg/ml in 50 mM Tris-Cl, pH 7.5, supplemented with 50 mM glucose. Fatty acid stocks were dissolved with co-solvent DMSO (1% final concentration in the reaction). To each reaction, 0.2 mM FAD, 5 mM DTT and 5 mM NADH were added. These chosen conditions lead to an FAD reducing environment which has been shown to be beneficial as Ohy reportedly is more active in the presence of reduced FADH_2_ compared with oxidised FAD (Engleder et al. 2015; Demming et al. 2017). Reactions (500 μl) were started by substrate addition (500 μM) and were carried out with orbital shaking (800 rpm) at 30 °C for 6 days (Demming et al. 2017; Engleder et al. 2019). All reactions were performed in triplicate. Additionally, each reaction was flushed with N_2_ to avoid oxidation of unsaturated fatty acids.

### *Rp*Ohy characterisation

For the standard small-scale biotransformations with purified enzyme, *Rp*Ohy (5 μM) was diluted in 20 mM acetate buffer, pH 5 (200 μl final volume) and 20 μM FAD was added. Reactions were started by substrate addition (500 μM oleic acid with 1% DMSO) and run for 5 h at 25 °C with orbital shaking (800 rpm). The temperature optimum was determined in the range of 20 °C to 60 °C with reactions run in acetate buffer (20 mM, pH 5). The pH optimum was investigated in the pH range of 3.6 to 10 at 25 °C. The following buffers were used (20 mM): pH 3.6–5 (acetate buffer), pH 5–6 (citrate buffer), pH 6–7.5 (potassium phosphate buffer), pH 7.2–8 (Tris-Cl buffer) and pH 8–10 (carbonate-bicarbonate buffer).

### Development of a novel oleate hydratase enzyme kinetic assay

In order to determine *Rp*Ohy kinetic parameters, a novel coupled assay was developed based on the ability of an alcohol dehydrogenase (ADH) to oxidise the product 10-hydroxystearic acid with concomitant reduction of NAD^+^ to NADH. A commercially available screen of ten different NAD^+^-dependent ADHs (Evoxx, Monheim am Rhein, Germany), supplied as lyophilised powders, was used to identify enzymes capable of oxidising 10-hydroxystearic acid. The following reaction conditions were used: 0.47 mg/ml enzyme (lyophilised powder), 2.5 mM 10-hydroxystearic acid, 2 mM NAD^+^, 20 mM PIPES buffer pH 6.5 and 10% DMSO. Initial rates of formation of NADH were measured spectrophotometrically at 340 nm using a 96-well plate reader (BioTek) and using ε_340_(NADH) = 6.2 mM^−1^ cm^−1^. The most active ADH under these conditions (ADH010) was used to validate the coupled assay with the well-established Ohy from *Elisabethkingia meningoseptica* (*Em-*OAH1) using the following conditions: 0–0.5 mg/ml ADH010, 0–2 mg/ml *Em-*OAH1, 2.5 mM oleic acid, 2 mM NAD^+^, 50 mM PIPES buffer pH 6.5 and 10% DMSO. Successful conditions for the coupled Ohy-ADH assay were subsequently applied to determine the kinetic parameters of *Rp*Ohy. The coupled assay conditions were the following: 200 μl reaction mixture containing 0.125–2.5 mM oleic acid, 2 mM NAD^+^, 50 mM PIPES pH 6.5 and 10% DMSO. The reactions were started by the addition of 0.3–1.5 μg/ml *Rp*Ohy and 0.01–0.5 mg/ml ADH010. The reactions were followed spectrophotometrically at 340 nm for 10 min. The initial rates were plotted versus the oleic acid concentration and apparent kinetic parameters were determined using the Hill equation for enzyme kinetics.

### Sample derivatisation

All reactions were quenched by the addition of 20 μl of 1 M HCl after the respective reaction time. Reactions were extracted with an equal ethyl acetate (EtOAc) volume before derivatisation. A total of 100 μl of extraction mixture was derivatised with 200 μl of derivatising agent (1:1 pyridine:*N,O*-bis(trimethylsilyl)trifluoroacetaminde (BSTFA)) with 1% trimethyl-silylchloride (TMSCl) for 1 h at 60 °C.

### Sample analysis

Achiral GC-FID analysis of the derivatised hydroxylated fatty acids was performed with a Shimadzu type GC-2014 equipped with a CP-Sil5 CB column (50 m × 0.53 mm × 1.0 μm) using N_2_ as carrier gas. The following conditions were used for the achiral separation using direct injection: injector 340 °C, detector (FID) 360 °C, column flow rate 20.0 ml/min, temperature program: start at 130 °C, hold time 4 min, rate 15 °C/min to 330 °C hold time 5 min.

Gas chromatography-mass spectrometry of derivatised hydroxylated fatty acids was performed with the Shimadzu GC-2010 system which is connected to the GCMS-QP2010s mass detector from Shimadzu. The column CP-Sil5 CB (25 m × 0.25 mm × 0.4 μm) was used. Injections were performed with the autoinjector AOC-20i from Shimadzu. The injector temperature was kept at 250 °C. The injector was used in split-mode with a split ratio of 30:1 at a pressure of 51.2 kPa. The temperature program for fatty acids 1 and 3-15: start at 130 °C, hold time 4 min, rate 15 °C/min to 330 °C hold time 5 min; temperature program for fatty acid 2: start at 130 °C, hold time 4 min, rate 5 °C/min to 325 °C hold time 7 min. Structure determination was based on the comparison of monomer peaks using external standards.

#### Databases

Bioproject accession number PRJNA555451. GenBank accession numbers MN563120 and MN563121.

## Results

### Extending the data set of an Orthologous Matrix (OMA) algorithm to identify novel biocatalysts

In the course of identifying novel hydratases with interesting properties in the genus *Rhodococcus*, an Orthologous Matrix (OMA) algorithm approach was used combining a large number of *Rhodococcus* strains from different families. The strain *R. pyridinivorans* DSM 20415 showed interesting behaviour towards α,β-unsaturated Michael acceptors in previous studies (Busch et al. 2020). As no genomic sequence data was available, it was decided to sequence the whole genome to subsequently incorporate this interesting strain into the generated Orthologous Matrix algorithm to further investigate whether this strains also produces an Ohy. The strain *R. pyridinivorans* DSM 20415 was originally isolated from activated sludge from a wastewater treatment plant (Mimura et al. 1969a, b).

The genome shows a size of 5,275,644 bp with a GC content of 67.7%. More detailed information on the sequence can be found in the Supplementary Information (Table S1), and the complete sequencing data is available at NCBI under bioproject accession number PRJNA555451. Protein and amino acid sequence of *Rp*Ohy are given in the Supplementary information.

### Orthologous Matrix algorithm analysis of oleate hydratases in the genus *Rhodococcus*

High-quality whole-genome sequences (WGS) from 43 *Rhodococcus* strains were either obtained by sequencing (in the case of *R. pyridinivorans* DSM 20415) or were publicly available from the National Centre for Biotechnology Information (NCBI) (Geer et al. 2009). Whole-genome sequences were analysed with the Orthologous Matrix (OMA) software (standalone version 2.2.0) using an improved matrix algorithm to generate pairwise orthologues of the proteomes of the annotated genomes (Altenhoff et al. 2013; Kumar et al. 2016). Within the 43 investigated strains, 20 oleate hydratases were identified. In total, three different HFams were recognised by the ‘Hydratase Engineering Database’: HFam 1, 2 and 3 (Schmid et al. 2016).

All investigated *R. equi* strains display an oleate hydratase from HFam 1. The largest group consists of 11 strains (including all *R. erythropolis* and all *R. qingshengii*, *R. enclensis* NIO-1009, *R. rhodochrous* DSM 101666 and *R. rhodochrous* ATCC 17895 as well as *Rhodococcus* R312) displaying an oleate hydratase from HFam 3. On the other hand, all *R. pyridinivorans* strains as well as the *R. biphenylivorans* TG9 show an oleate hydratase from HFam 2. Interestingly, one strain, *R. erythropolis* DSM 43066, displays two oleate hydratases with one belonging to HFam 2 and the other one to HFam 3 (Table S2).

**Fig. 1:**
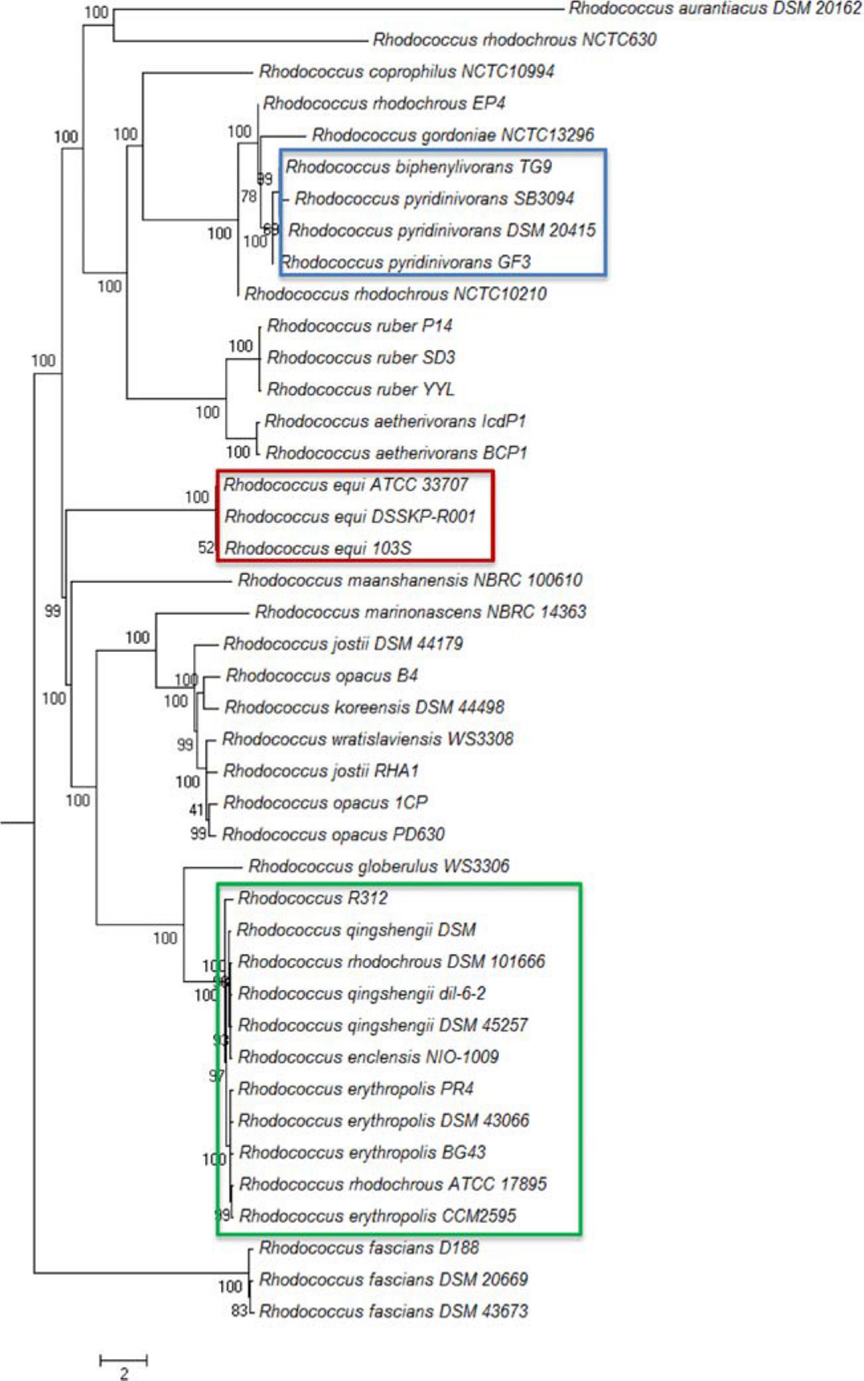
Phylogenetic tree of 43 investigated *Rhodococcus* strains based on the 1% of most complete computed orthologous groups (Kumar et al. 2016; Train et al. 2017). HFam 2 or ‘*R. pyridinivorans*’-clade highlighted in blue, HFam 1 or ‘*R. equi*’-clade in red and HFam 3 or ‘*R. erythropolis*’-clade in green

To investigate the relation between the strains, a phylogenetic tree was created based on the 1% of most complete computed orthologous groups and visualised with MEGA (Fig. 1) (Kumar et al. 2016; Train et al. 2017). Similar results were obtained when a phylogenetic tree was based on the 16S rRNA sequences (Fig. S1). As clearly visible, the three groups inheriting the same type of oleate hydratase cluster together. They form three independent clades from now on called ‘*erythropolis*-clade’ (green), ‘*pyridinivorans*-clade’ (blue) and ‘*equi*-clade’ (red).

To analyse the relation between the annotated oleate hydratases amongst the different families, a phylogenetic tree was generated (Fig. S2). *R. erythropolis* DSM 43066 is the only strain with two different types of oleate hydratase present. While the ‘*R. erythropolis*’- and ‘*R. equi*’-clade are built up uniformly, the oleate hydratase from *R. erythropolis* DSM 43066 forms its own sub-group within the ‘*R. pyridinivorans*’-clade. To identify similarities and distinct differences between the three oleate hydratase groups, a sequence alignment was performed to study the specific conservation pattern.

### Fatty acid substrate specificity of *Re*Ohy (HFam 3) and *Rp*Ohy (HFam 2)

A number of fatty acid substrates were tested for activity using *E. coli* whole cells overexpressing *Rp*Ohy (DSM 20415), *Re*Ohy (PR4) as well as *E. coli* TOP10 cells containing an empty pBAD/His A expression vector as negative control. Due to recent reports about the benefits of applying whole-cell systems over cell-free extract or purified enzymes such as a higher operational stability, the prevention of time-consuming steps like protein purification and therefore an overall easier handling, all reactions were carried out in a whole-cell system (Demming et al. 2017; Engleder et al. 2019). All reactions using *E. coli* TOP10 cells bearing the empty pBAD/His A vector did not show any product formation ruling out background hydration activity. Fifteen fatty acids were tested with representatives from *R. pyridinivorans* DSM 20415 (*Rp*Ohy, HFam 2) and from *R. erythropolis* PR4 (*Re*Ohy (PR4), HFam 3, Table 1, molecular structures Table S3). If applicable, these whole-cell screening results were compared with the earlier described *Re*Ohy (CCM2595, HFam 3) substrate screening using purified enzyme by Lorenzen et al. (2017).

**Table 1:** Tested substrates and position of hydroxyl group in product of *Rp*Ohy (DSM 20415) and *Re*Ohy (PR4) whole-cell substrate screening. All reactions were performed in triplicate

Entry	Substrate		*Rp*Ohy^a^	*Re*Ohy (PR4)^a^	*Re*Ohy (CCM2595)^b^
1	Myristoleic acid	14:1, *cis*-9	10	10	--^c^
2	Palmitoleic acid	16:1, *cis*-9	10	10	10
3	Oleic acid	18:1, *cis*-9	10	10	10
4	Linoleic acid	18:2, *cis*-9,12	10	10	10
5	Pinolenic acid	18:3, *cis*-5,9,12	10	10	Unknown^d^
6	*cis*-Vaccenic acid	18:1, *cis*-11	--^c^	--^c^	--^c^
7	*trans*-Vaccenic acid	18:1, *trans*-11	--^c^	--^c^	unknown^d^
8	γ-Linolenic acid	18:3, *cis*-6,9,12	10	10	10
9	Ricinoleic acid	18:1, *cis*-9, (*R*)-12-OH	10	--^c^	unknown^d^
10	*cis*-11-Eicosenoic acid	20:1, *cis*-11	12	12	unknown^d^
11	*cis*-8,11-Eicosadienoic acid	20:2, *cis*-8,11	--^c^	12	unknown^d^
12	*cis*-8,11,14-Eicosatrienoic acid	20:3, *cis*-8,11,14	--^c^	12	--^c^
13	*cis*-5,8,11,14-Eicosatetraenoic acid (arachidonic acid)	20:4, *cis*-5,8,11,14	--^c^	12	--^c^
14	Erucic acid	22:1, *cis*-13	--^c^	14	unknown^d^
15	Nervonic acid	24:1, *cis*-15	--^c^	--^c^	unknown^d^

^a^Position of hydroxyl group determined by GC-MS.

^b^Position of hydroxyl group and substrate acceptance determined by Lorenzen et al. (2017)

^c^No water addition

^d^Not tested

Three substrates were converted by neither of the tested Ohys: nervonic acid as well as *cis*- and *trans*-vaccenic acid. Myristoleic acid was converted by both *Re*Ohy (PR4) and *Rp*Ohy (DSM 20415) under the chosen whole-cell conditions. While *Re*Ohy (PR4) did not show any activity towards ricinoleic acid, *Rp*Ohy produced the 10,12-dihydroxylated fatty acid. Fatty acids 10–13 all carry a *cis*-double bond in 11-position as does *cis*-vaccenic acid. While *Rp*Ohy only converted *cis*-11-eicosenoic acid (10) with low activity, *Re*Ohy (PR4) showed activity towards all four long, unsaturated fatty acids leading to the mono-hydrated 12-hydroxy fatty acids, exclusively. *Re*Ohy (PR4) is therefore, to our knowledge, the first Ohy being able to selectively catalyse the water addition to long-chain, unsaturated fatty acids selectively in 12-position. Chromatograms comparing conversions of *Rp*Ohy and *Re*Ohy (PR4) as well as the GC-MS data of hydroxylated fatty acids can be found in the Supplementary Information (Figs. S6-S29).

### *Rp*Ohy (DSM 20415) characterisation

The Ohy from *R. pyridinivorans* DSM 20415 has a calculated protein weight of 67.8 kDa and consists of 601 amino acids. After heterologous expression in *E. coli* TOP10 cells, the N-terminally His6-tagged enzyme was purified by Ni^2+^-affinity chromatography and a subsequent size-exclusion chromatography (SEC). SEC revealed the presence of multiple oligomeric states, predominantly monomeric and dimeric with additional larger aggregates (> 10 meric) whose ratios were shown to be independent on the enzyme concentration used (Fig. S30 and Table S4). The purified protein had a yellow colour indicating that the FAD cofactor was incorporated in the enzyme after the purification. The standard activity of *Rp*Ohy was measured by following the conversion of oleic acid to 10-hydroxystearic acid for 5 h.

**Fig. 2:**
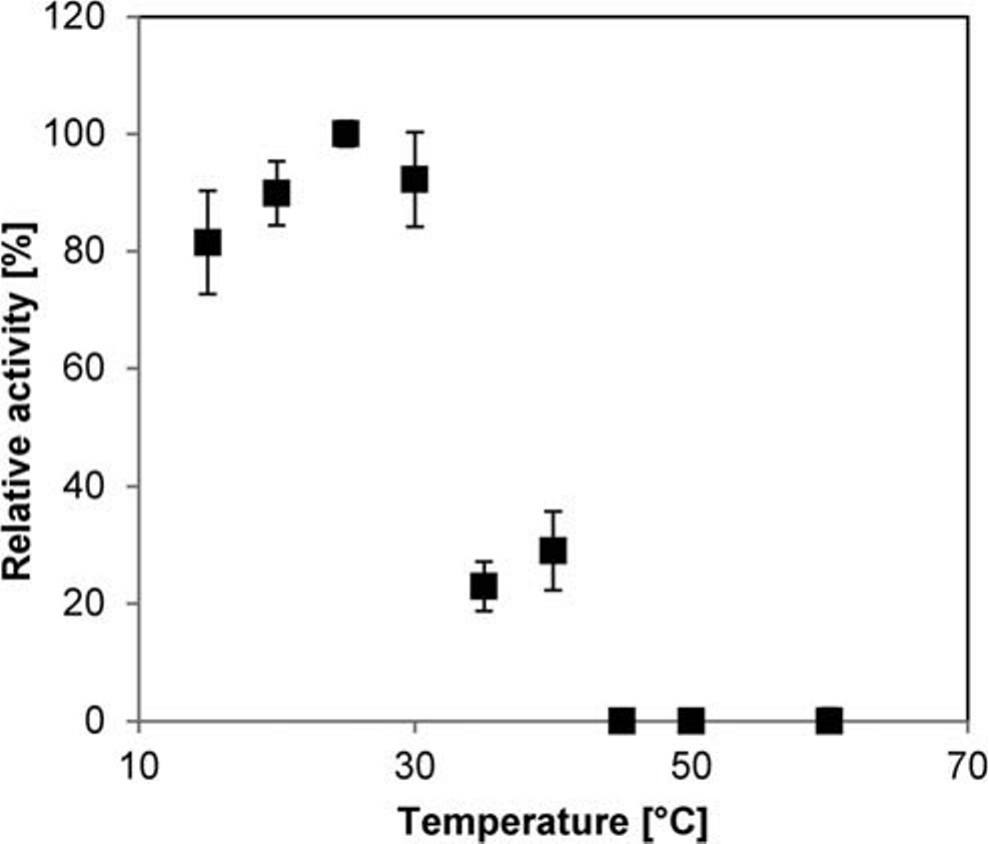
Temperature tolerance with temperature optimum of *Rp*Ohy. Reactions (triplicates) were carried out in 20 mM acetate buffer (pH 5) for 5 h at the respective temperatures. Reaction conditions: 5 μM purified *Rp*Ohy, 500 μM oleic acid with 1% DMSO as co-solvent and 20 μM FAD. Relative activity is based on the highest absolute activity (here at 25 °C) and set to be 100%

A beneficial effect of FAD addition to the reaction mixture was investigated in the range of 0–100 μM. Even without any addition of FAD, product formation (35%) was observed, but the addition of FAD increased the activity. However, the amount of added FAD (10–100 μM) did not have a significant impact on the product formation (51–57%) (Figs. S31 and S32). Therefore, it was decided to add 20 μM to ensure a fully FAD-saturated protein in all bioconversions. The temperature tolerance for *Rp*Ohy was measured in a range from 15 to 60 °C (Fig. 2). The highest activity was achieved at 25 °C, but the protein remained active in a broad temperature range from 15 °C to 40 °C. Higher temperatures, however, led to its deactivation.

**Fig. 3:**
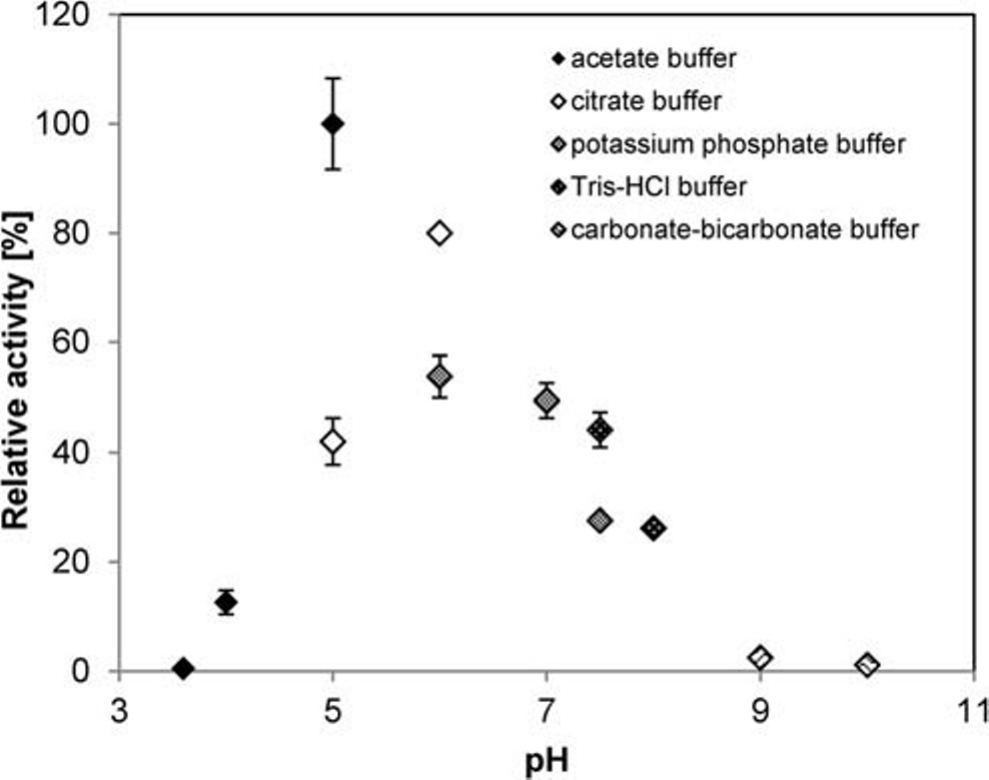
pH tolerance with pH optimum of *Rp*Ohy. Reactions (triplicates) were carried out at 25 °C for 5 h at the respective pH values. Reaction conditions: 5 μM purified *Rp*Ohy, 500 μM oleic acid with 1% DMSO as co-solvent and 20 μM FAD. Relative activity is based on the highest absolute activity (here in 20 mM acetate buffer, pH 5) and set to be 100%

The pH acceptance of *Rp*Ohy was investigated in the pH range of 3.6–10 (Fig. 3). The highest activity was determined at pH 5 (acetate buffer, 20 mM). The protein remained active over a broad pH range from pH 5–8.

### Development of a novel sensitive oleate hydratase kinetic assay

**Fig. 4:**
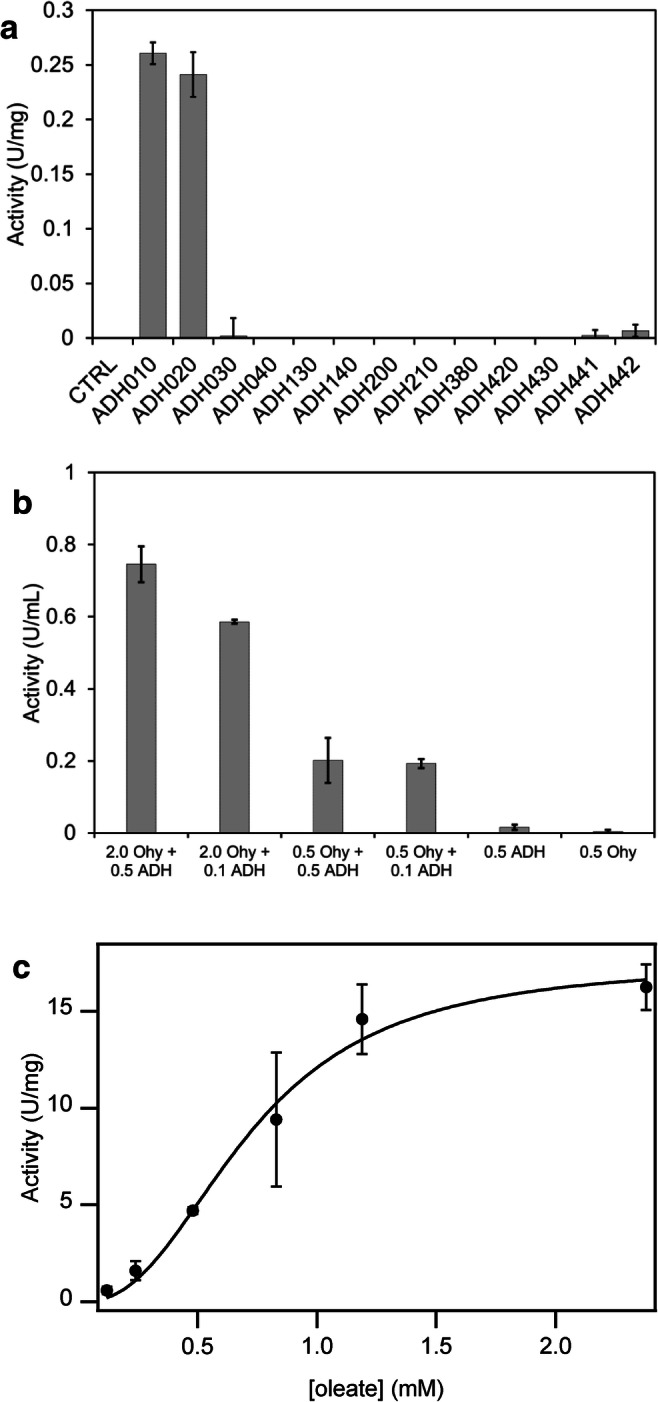
Assay development and kinetics of *Rp*Ohy. **a** Screening of ADHs for the coupled assay of Ohy catalyzed conversion of oleic acid. Reaction conditions: 0.47 mg/ml ADH (lyophilised powder), 2.5 mM 10-hydroxystearic acid, 2 mM NAD^+^, 20 mM PIPES buffer pH 6.5 and 10% DMSO. **b** Validation of the Ohy-ADH coupled assay using *Em-*OAH1. Enzyme amounts below the horizontal axis are given in mg/ml protein for *Em-*OAH1 and mg/ml lyophilised powder for AHD010. Reaction conditions: 0–0.5 mg/ml ADH010, 0–2 mg/ml *Em-*OAH1, 2.5 mM oleic acid, 2 mM NAD^+^, 50 mM PIPES buffer pH 6.5 and 10% DMSO. **c** Apparent cooperative kinetics of *Rp*Ohy measured in a coupled assay with ADH010. Assay conditions: 0.125–2.5 mM oleic acid, 2 mM NAD^+^, 50 mM PIPES pH 6.5 and 10% DMSO, 0.3–1.5 μg/ml *Rp*Ohy and 0.01–0.5 mg/ml ADH010. The solid line is a fit to the cooperative kinetics Hill equation ${V}_0=\frac{V_{\mathrm{max}}\cdotp {\left[S\right]}^n}{K_{0.5}^n+{\left[S\right]}^n}$ with *K*_0.5_ = 0.72 ± 0.07 mM and *V*_max_ = 17.5 ± 1.4 U/mg, *n* = 2.45 ± 0.47

In order to determine the kinetic parameters of *Rp*Ohy, a novel assay was established to allow sensitive and straightforward rate measurements in the sub-mM substrate concentration range. The assay is based on the coupling of the hydration of oleic acid to 10-hydroxystearic acid by Ohy with the subsequent oxidation of 10-hydroxystearic acid to 10-ketostearic acid by an NAD^+^-dependent ADH, which allows the spectrophotometric measurement of NAD^+^ reduction. A commercial screen of ten different NAD^+^-dependent ADHs was used and two enzymes, ADH010 and ADH020, which efficiently oxidise 10-hydroxystearic acid, were identified (Fig. 4a).

The most efficient enzyme, ADH010, was subsequently used to validate the coupled assay with the canonical Ohy from *Elisabethkingia meningoseptica* (*Em-*OAH1) (Fig. 4b). It was found that a concentration of 0.1 mg/ml ADH010 (based on the weight of the lyophilised powder) was sufficient to detect the activity of 0.5 mg/ml (protein concentration) *Em-*OAH1, but was limiting the activity at 2 mg/ml protein. By using a five times higher ADH concentration, the activity of both 0.5 and 2 mg/ml *Em-*OAH1 was measured successfully. Control measurements with only ADH or only *Em-*OAH1 showed no to minor background activity.

The kinetic parameters of *Rp*Ohy with oleic acid as substrate were determined using the novel coupled assay. In order to ensure the ADH activity was not limiting in the coupled assay, a large excess of ADH was used, and the *Rp*Ohy concentration was at least 300-fold lower than the *Em-*OAH1 concentration that was used for the validation of the assay. The kinetic data followed a cooperative kinetics model with a *K*_0.5_ value of 0.72 ± 0.07 mM, a *V*_max_ of 17.5 ± 1.4 U/mg and a Hill coefficient *n* of 2.45 ± 0.47 (Fig. 4c).

## Discussion

### Diversity of Ohys in the genus *Rhodococcus*

Genome analysis showed sequence diversity among Ohys that can be exploited for the discovery of novel features and will broaden future applications. *Rhodococcus* cells host a large number of interesting catabolic enzymes and are known for their extraordinary potential for bioremediation (Kim et al. 2018; Busch et al. 2020; Busch et al. 2019). The genus *Rhodococcus* was investigated in detail for the presence of oleate hydratases using an Orthologous Matrix algorithm. From the analysis of 43 whole-genome sequences, 20 putative Ohys were identified clustered into three different clades. Sequence alignment and phylogenetic analysis revealed that each clade contained oleate hydratases from one specific family (HFam 1–3). Family-specific amino acid patterns were elucidated.

Recently, Schmid et al. described 80 highly conserved amino acids throughout all investigated Ohys (Schmid et al. 2016). While the function of some conserved amino acids is already known, others remain unknown. Up until now, all Ohys require FAD for a successful substrate conversion although the redox state of the cofactor is not changed during the reaction (Engleder and Pichler 2018). Currently, the scientific consensus is that the FAD cofactor only plays a structural role being essential for a correct positioning of amino acids in the active site (Engleder et al. 2015; Lorenzen et al. 2017). One sensitive sequence motif for the FAD cofactor binding region was identified as G69xG[LI][AG]x[LM][AS][AG]Ax[FY][LM][IV]R[DE][GA]x(3)Gxx[IV]x[IFVl][LFY]E96 (positions according to *Em-*OAH1) (Schmid et al. 2016). While all identified Ohys from HFam 1 (G14-E41) and HFam 3 (G13-G40) as well as HFam 2 Ohy from *R. erythropolis* DSM 43066 (G29-E56) share the same motif, the other Ohys in the ‘*pyridinivorans*’-clade differ distinctively in two of the conserved positions: T33 (instead of A (79%) or G (11%) (Schmid et al. 2016)) and S37 (instead of A (71%), G (16%) or T (8%)) (positions 73 and 77 according to *Em-*OAH1)(Schmid et al. 2016) (Fig. S3). Especially threonine (T33) instead of Ala or Gly will increase the polarity. Additionally, amino acids R118-M123 (*Em-*OAH1) placed in a loop-region were shown to be involved in cofactor binding and catalysis (Engleder et al. 2015). While the HFam 2 clade shows a ‘RGGREM’ motif like *Em-*OAH1 (HFam 11, Fig. S4), *Rhodococcus* HFam 1 and HFam 3 Ohys all share a ‘RGGRML’ motif. In *Em-*OAH1, E122 takes an important role in the water activation step (Engleder et al. 2015) and based on the high motif similarity, it is likely that *Rhodococcus* HFam 2 Ohys react in a similar way. The other two groups, however, do not share the same motif and previous studies claimed a different reaction mechanism for HFam 3 Ohys (Lorenzen et al. 2017). Due to the same amino acid pattern, it is likely that *Rhodococcus* HFam 1 Ohys will react in the same way as HFam 3 Ohys.

Finally, residues 436 and 438 (numbering according to *Em-*OAH1) are involved in the binding of the carboxylate function of the substrate (Engleder et al. 2015; Lorenzen et al. 2017). At both positions, the probabilities of four different amino acids were described to be 54% T, 31% V, 8% A and 5% S (residue 436 *Em-*OAH1) and 48% N, 22% H, 12% A and 9% P (residue 438 *Em-*OAH1) (Schmid et al. 2016). Analysis of the two residues showed a distinct pattern depending on the chosen HFam (Fig. S5). While *Rhodococcus* Ohys from HFam 1 all show the combination of residues valine and alanine, Ohys in the *pyridinivorans*-clade all exhibit residues threonine and asparagine, respectively. HFam 3, on the other hand, displays amino acids valine and histidine. The latter residue was shown to be directly involved in the carboxylate binding in the case of *Em-*OAH1 as a single-point mutation from asparagine to alanine (N438A according to *Em-*OAH1) led to a reduced activity (Engleder et al. 2015). The presence of alanine in that position in the HFam 1 Ohys additionally hints at a different reaction mechanism for this group of enzymes. A recent study investigated the impact of these two amino acids on the final regio- and stereoselectivity and showed that selective mutations can lead to a complete reversal of selectivity with the example of the two fatty acid hydratases from *Lactobacillus acidophilus* (FA-HY1 and FA-HY2). Here, less bulky substituents in these two positions enabled the substrate to go deeper into the carboxylate end of the substrate channel (Eser et al. 2020).

The family-specific patterns could hint at different reaction mechanisms and explain the differences in substrate recognition. Docking studies with structural models based on published crystal structures from *L. acidophilus* NCFM for *Rp*Ohy and *Re*Ohy (CCM2595) for *Re*Ohy (PR4) were inconclusive due to the absence of FAD in the crystal structures which resulted in uncertainty of the position and configuration of the cofactor. The structure elucidation of *Rp*Ohy will give further insight in the HFam 2-specific reaction mechanism as well as further explain the differences in substrate acceptance.

### *Rp*Ohy and *Re*Ohy (PR4) exhibit a complementary substrate scope

Two representatives from HFam 2 and HFam 3, *Rp*Ohy and *Re*Ohy (PR4), were chosen for heterologous expression in *E. coli* and tested on a large number of fatty acids using whole-cell biotransformations. When using the purified enzyme with shorter incubation times, no water addition was observed by Lorenzen et al. for *Re*Ohy (CCM2595) (Lorenzen et al. 2017). This example further shows the benefits of the more stable whole-cell system over the use of purified enzyme.

The two tested Ohys were found to act complementary with longer and sterically more demanding fatty acids. As expected, fatty acids palmitoleic acid and oleic acid were converted by both tested enzymes. Linoleic acid and γ-linolenic acid were also accepted by all Ohys, and water addition was exclusively observed in 10-position. The results from this screening are in alignment with the results obtained by Lorenzen et al. for *Re*Ohy (CCM 2595) (Lorenzen et al. 2017). The all-*cis*-configurated pinolenic acid (18:3, *cis*-5,9,12) was a substrate for both tested *Rhodococcus* Ohys. Here, the water addition was only observed in 10-position. Up to now, only two other Ohys from *Lactobacillus acidophilus* (NTV001 (FA-HY1) and LMG 11470) were shown to convert pinolenic acid whereby both catalyse the water addition in 13-position (Engleder et al. 2015; Hirata et al. 2015; Kim et al. 2015). This shows a clear preference for the 10-position over the 13-position for both *Rhodococcus* Ohys which can be explained by the two amino acids V393 and H395 (*Re*Ohy (PR4)) and T390 and N392 (*Rp*Ohy) (corresponding to T436 and N438 in *Em-*OAH1) following the results from recent studies (Eser et al. 2020). In FA-HY1, these positions are occupied by two smaller serine amino acids meaning that the substrate is entering the substrate tunnel further leading to the water addition in 13-position (Eser et al. 2020). In our example, however, histidine and asparagine are bulkier thereby directing a water addition into 10-position. In general, the addition in 13-position was not detected with any of the tested substrates.

The tendency to convert large fatty acids could be explained by a larger active site in *Re*Ohy (PR4). In this screening, *Re*Ohy (PR4) was shown to even convert erucic acid in 14-position which, to our knowledge, has not been reported for any other Ohy. The active state as a monomer might have an influence on the ability to convert longer fatty acids. Surprisingly, both Ohys were not able to convert *cis*-vaccenic acid which has significant similarities with *cis*-11-eicosenoic acid but is two carbon atoms shorter. The absence of these two carbon atoms is most likely the reason why the shorter *cis*-vaccenic acid is not accepted. Nervonic acid (24:1, *cis*-15) is possibly too long to fit in the active site of the proteins and *trans*-vaccenic acid does not reach the active site as the double bond is required to be *cis*-configurated as was reported earlier (Demming et al. 2018; Engleder and Pichler 2018).

Next to *Rp*Ohy, only two other hydratases (*Lactobacillus plantarum* AKU 1009a and *Lysinibacillus fusiformis*) were reported to show activity towards ricinoleic acid (Seo et al. 2013; Takeuchi et al. 2015). Its interesting emulsifying properties make 10,12-dihydroxystearic acid a potentially useful biosurfactant (Seo et al. 2013). FA-HY1 (*Lactobacillus acidophilus*) was described to also convert fatty acids 12 and 13. However, the water addition for fatty acid 13 occurred in 15-position while with fatty acid 12, water addition took place in 12- and 15-position (Hirata et al. 2015).

This comprehensive substrate screening gives new insight into the capability of Ohys from *Rhodococcus* to convert a number of fatty acids selectively. It was shown that especially with more complex and unsaturated fatty acids, both investigated Ohys act complementary which can be further exploited for industrial applications. The described variety of the substrate scope of investigated Ohys is therefore consistent with the sequence variety among the different oleate hydratase families and members.

### *Rp*Ohy is a novel oleate hydratase

*Rp*Ohy is active over a broad pH range (pH 5–8) with an optimum at circa pH 6.5 and temperature range (15–40 °C) with an optimum at circa 25 °C, which is comparable with the results obtained by Lorenzen et al. for *Re*Ohy (CCM 2595) who described a temperature optimum at 28 °C and a pH optimum of 7.2 with good conversion rates in the range from pH 5–8 (Lorenzen et al. 2017).

The kinetics of *Rp*Ohy showed a significant cooperative effect, which has rarely been reported for Ohys. Only recently for *Staphylococcus aureus* Ohy, similar cooperative kinetics was reported with a similar Hill coefficient of 2.2 ± 0.3 (Subramanian et al. 2019). Most Ohys have been reported to display regular Michaelis-Menten kinetics with *K*_M_ in the range from 0.1 to 0.6 mM and *V*_max_ from 0.6 to 4 U/mg (Bevers et al. 2009; Volkov et al. 2010; Rosberg-Cody et al. 2011; Joo et al. 2012a; Kim et al. 2012; Joo et al. 2012b). The reported values for *Re*Ohy (CCM 2595) are *K*_M_ = 0.49 mM and *V*_max_ = 1.27 U/mg (Lorenzen et al. 2017). The kinetic parameters for *Rp*Ohy suggest that this HFam 2 enzyme displays apparent cooperative kinetics and has a higher *V*_max_ than *Re*Ohy (CCM 2595). However, due to the technical challenges of measuring the rate of conversion of a barely soluble substrate to an insoluble product, the activity measurements are strongly dependent on the precise conditions that have been used. It is therefore difficult to directly compare the parameters with the reported values for other Ohys that have been measured with different experimental conditions. Consequently, it is possible that the observed apparent cooperative kinetics does not reflect an allosteric effect on the enzyme by the substrate, but rather concentration-dependent substrate availability under the conditions that were used. Furthermore, the observed saturation of the activity at high substrate concentration may be affected by the critical micelle concentration of oleic acid.

### A novel oleate hydratase enzyme assay

ADH010 has been reported to reduce 3-nonanone and AHD020 has been reported to reduce ethyl pyruvate by the enzyme manufacturer. Both enzymes have been reported to be inactive for the oxidation of *rac*-phenylalaninol (amino alcohol) and the reduction of 2-azidoacetophenone (azido ketone) (Mestrom et al. 2017; Schrittwieser et al. 2013). Interestingly, the reduced form of 3-nonanone, i.e. 3-nonanol, and 10-hydroxystearic acid share some resemblance as these are both secondary alcohols in a long aliphatic chain. Any reduced form of ethyl pyruvate does not have any apparent similarity to 10-hydroxystearic acid, which may imply that ADH020 has a broad substrate scope.

Secondary alcohol dehydrogenases that can oxidise 10-hydroxystearic acid to 10-ketostearic acid from *Pseudomonas* sp. NRLL B-3266 and from *Nocardia cholesterolicum* NRRL 5767 have been reported previously (Huang et al. 2020; Niehaus et al. 1978). Although preliminary reports have shown that cascade reactions with Ohy and ADHs are possible, these have never been used to develop an efficient Ohy activity assay (Koritala et al. 1989; Song et al. 2013). The ability to use NADH production to report Ohy activity is highly desired as it facilitates high throughput screening for Ohy activity and conversely enables directed evolution and other protein engineering approaches that are dependent on considerable screening efforts. We have successfully shown that the coupled assay can be used to measure the activity of *Em-*OAH1, *Rp*Ohy and most likely any Ohy. The method can be readily extended to other fatty acid hydratase substrates by searching for other optimal ADHs. Furthermore, the novel coupled assay will enable more extensive kinetic investigation of Ohys under many different conditions.

## Electronic supplementary material

ESM 1 (PDF 696 kb).
