# Supplementary material for: Exploring the abundance of oleate hydratases in the genus Rhodococcus—discovery of novel enzymes with complementary substrate scope
Source: Appl Microbiol Biotechnol. 2020 May 1;104(13):5801–12. doi: 10.1007/s00253-020-10627-7 (PMC7306040; doi:10.1007/s00253-020-10627-7)
Supplement: Supplementary file 1 — (PDF 696 kb). [file 253_2020_10627_MOESM1_ESM.pdf]

Supplementary Information

Applied Microbiology and Biotechnology

Exploring the abundance of oleate hydratases in the genus *Rhodococcus* – discovery of novel enzymes with complementary substrate scope

Hanna Busch, Fabio Tonin, Natália Alvarenga, Marcel van den Broek, Simona Lu, Jean-Marc Daran, Ulf Hanefeld and Peter-Leon Hagedoorn*

Department of Biotechnology, Delft University of Technology, Van der Maasweg 9, 2629 HZ Delft, The Netherlands

Corresponding author: Peter-Leon Hagedoorn

Telephone: +31 (0)15 278 2334

Email: P.L.Hagedoorn@tudelft.nl

Contents

Table S1: Whole-genome sequencing data for *R. pyridinivorans* DSM 20415 1

Table S2: List of *Rhodococcus* strains containing an oleate hydratase categorised based on their HFam affiliation 1

Figure S1: Phylogenetic tree of 43 investigated *Rhodococcus* strains based on 16S rRNA sequences 2

Figure S2: Phylogenetic tree of discovered oleate hydratases from *Rhodococcus* showing three clades: (i) erythropolis-clade, (ii) equi-clade, (iii) pyridinivorans-clade. 3

Figure S3: CLUSTAL multiple sequence alignment of *Rhodococcus oleate* hydratases compared to *Em*-OAH1 in the range of conserved FAD-binding region (G69-E96) 4

Figure S4: CLUSTAL multiple sequence alignment of *Rhodococcus* oleate hydratases compared to *Em*-OAH1 in the area of conserved R118GGREM123 (*Em*-OAH1) motif 4

Figure S5: CLUSTAL multiple sequence alignment of *Rhodococcus* oleate hydratases compared to *Em*-OAH1 in the area of conserved amino acids involved in carboxylate binding (T436 and N438) 4

Table S3: Molecular structures of tested fatty acids 5

Figure S6: GC-MS chromatograms showing the products of *E. coli* whole-cell bioconversions of *Re*Ohy (black) and *Rp*Ohy (pink) of fatty acid #1 – myrestoleic acid. 6

Figure S7: Mass spectrum of silylated, hydroxylated myrestoleic acid 6

Figure S8: GC-MS chromatograms showing the products of *E. coli* whole-cell bioconversions of *Re*Ohy (black) and *Rp*Ohy (pink) of fatty acid #2 – palmitoleic acid 6

Figure S9: Mass spectrum of silylated, hydroxylated palmitoleic acid 7

Figure S10: GC-MS chromatograms showing the products of *E. coli* whole-cell bioconversions of *Re*Ohy (black) and *Rp*Ohy (pink) of fatty acid #3 – oleic acid. 7

Figure S11: Mass spectrum of silylated, hydroxylated oleic acid 7

Figure S12: GC-MS chromatograms showing the products of *E. coli* whole-cell bioconversions of *Re*Ohy (black) and *Rp*Ohy (pink) of fatty acid #4 – linoleic acid. 8

Figure S13: Mass spectrum of silylated, hydroxylated linoleic acid 8

Figure S14: GC-MS chromatograms showing the products of *E. coli* whole-cell bioconversions of *Re*Ohy (black) and *Rp*Ohy (pink) of fatty acid #5 – pinolenic acid 8

Figure S15: Mass spectrum of silylated, hydroxylated pinolenic acid 9

Figure S16: GC-MS chromatograms showing the products of *E. coli* whole-cell bioconversions of *Re*Ohy (black) and *Rp*Ohy (pink) of fatty acid #8 – γ-linolenic acid 9

Figure S17: Mass spectrum of silylated, hydroxylated γ-linolenic acid 9

Figure S18: GC-MS chromatograms showing the products of *E. coli* whole-cell bioconversions of *Re*Ohy (black) and *Rp*Ohy (pink) of fatty acid #9 – ricinoleic acid 10

Figure S19: Mass spectrum of silylated, hydroxylated ricinoleic acid 10

Figure S20: GC-MS chromatograms showing the products of *E. coli* whole-cell bioconversions of *Re*Ohy (black) and *Rp*Ohy (pink) of fatty acid #10 – *cis*-11-eicosenoic acid 10

Figure S21: Mass spectrum of silylated, hydroxylated *cis*-11-eicosenoic acid 11

Figure S22: GC-MS chromatograms showing the products of *E. coli* whole-cell bioconversions of *Re*Ohy (black) and *Rp*Ohy (pink) of fatty acid #11 – *cis*-11,14-eicosadienoic acid 11

Figure S23: Mass spectrum of silylated, hydroxylated *cis*-11,14-eicosadienoic acid 11

Figure S24: GC-MS chromatograms showing the products of *E. coli* whole-cell bioconversions of *Re*Ohy (black) and *Rp*Ohy (pink) of fatty acid #12 – *cis*-8,11,14-eicosatrienoic acid **12**

Figure S25: Mass spectrum of silylated, hydroxylated *cis*-11,14-eicosadienoic acid **12**

Figure S26: GC-MS chromatograms showing the products of *E. coli* whole-cell bioconversions of *Re*Ohy (black) and *Rp*Ohy (pink) of fatty acid #13 – arachidonic acid **12**

Figure S27: Mass spectrum of silylated, hydroxylated arachidonic acid **13**

Figure S28: GC-MS chromatograms showing the products of *E. coli* whole-cell bioconversions of *Re*Ohy (black) and *Rp*Ohy (pink) of fatty acid #14 – erucic acid **13**

Figure S29: Mass spectrum of silylated, hydroxylated erucic acid **13**

Figure S30: Size exclusion chromatography of *Rp*Ohy **14**

Table S4: Molecular weight estimation *Rp*Ohy **14**

Figure S31: Influence of FAD addition to biotransformation of oleic acid to 10-hydroxystearic acid **15**

Figure S32: UV-visible absorption spectrum of purified *Rp*Ohy in the range from 300 – 600 nm **15**

Nucleotide sequence *Rp*Ohy 16

Amino-acid sequence *Rp*Ohy 16

Nucleotide sequence synthetic *Rp*Ohy construct codon optimized for *E. coli* including his-tag 17

Amino-acid sequence synthetic *Rp*Ohy contstruct including histag 17

References 18

Table S1: Whole-genome sequencing data for *R. pyridinivorans* DSM 20415 (Aziz et al. 2008).

Genome Size [kb]	5,275,644
GC content [%]	67.7
N50	204034
L50	7
Number of contigs (> 1kbp)	174
Number of subsystems	426
Number of coding sequences	5018
Number of RNAs	67

Table S2: List of *Rhodococcus* strains containing an oleate hydratase categorised based on their HFam affiliation (Schmid et al. 2016).

HFam1	HFam2	HFam3
Rhodococcus equi 103S	*Rhodococcus biphenylivorans* TG9	*Rhodococcus* R312
Rhodococcus equi ATCC 33707	*Rhodococcus pyridinivorans* DSM 20415	*Rhodococcus enclensis* NIO-1009
Rhodococcus equi DSSKP-R001	*Rhodococcus pyridinivorans* GF3	*Rhodococcus erythropolis* BG43
	Rhodococcus pyridinivorans SB3094	*Rhodococcus erythropolis* CCM2595
	Rhodococcus erythropolis DSM 43066	*Rhodococcus erythropolis* PR4
		Rhodococcus erythropolis DSM 43066
		Rhodococcus qingshengii DSM 45257
		Rhodococcus qingshengii DSM 46766
		Rhodococcus qingshengii dil-6-2
		Rhodococcus rhodochrous DSM 101666
		Rhodococcus rhodochrous ATCC 17895

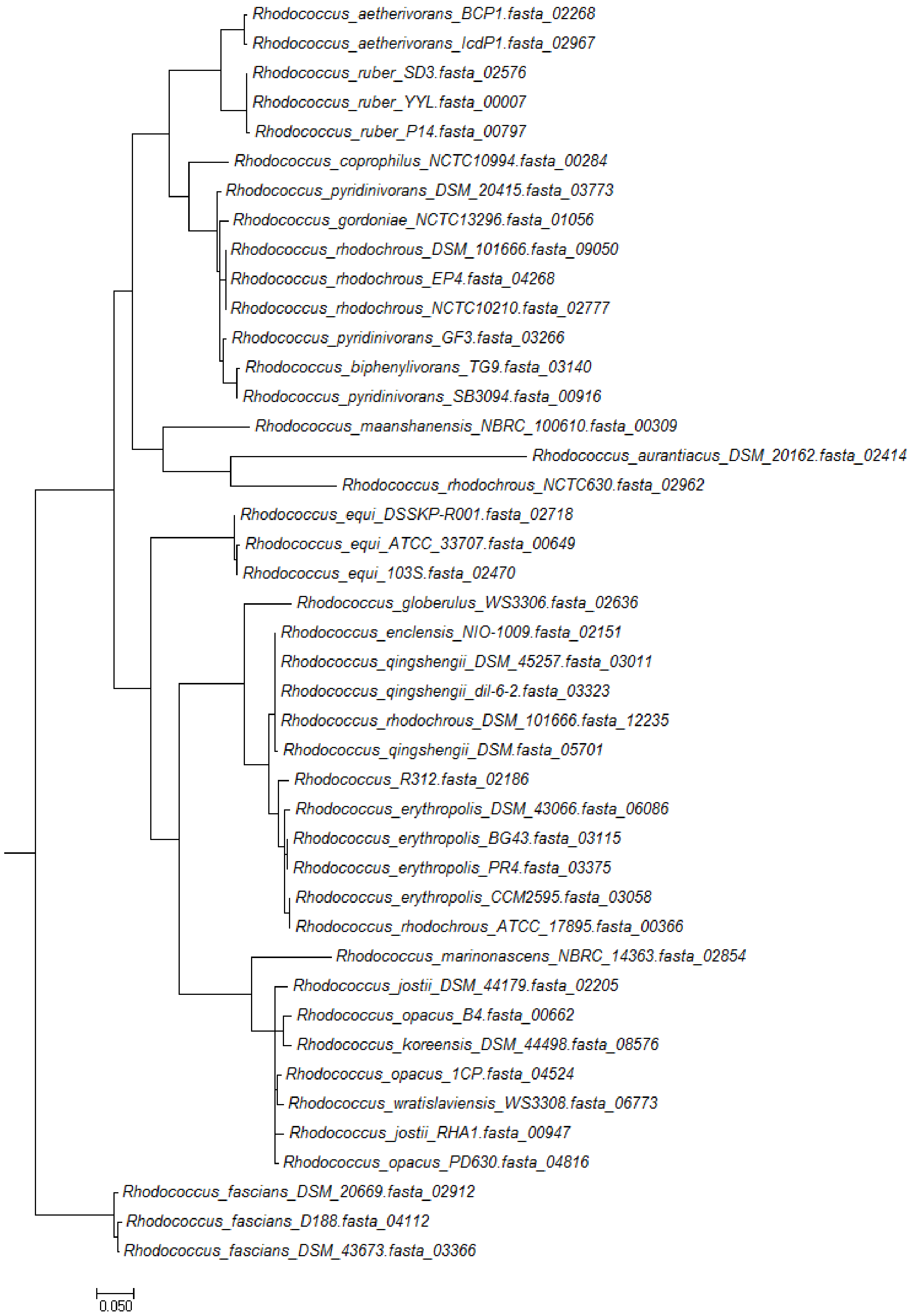

Figure S1: Phylogenetic tree of 43 investigated *Rhodococcus* strains based on 16S rRNA sequences (Edgar 2004; Price et al. 2010).

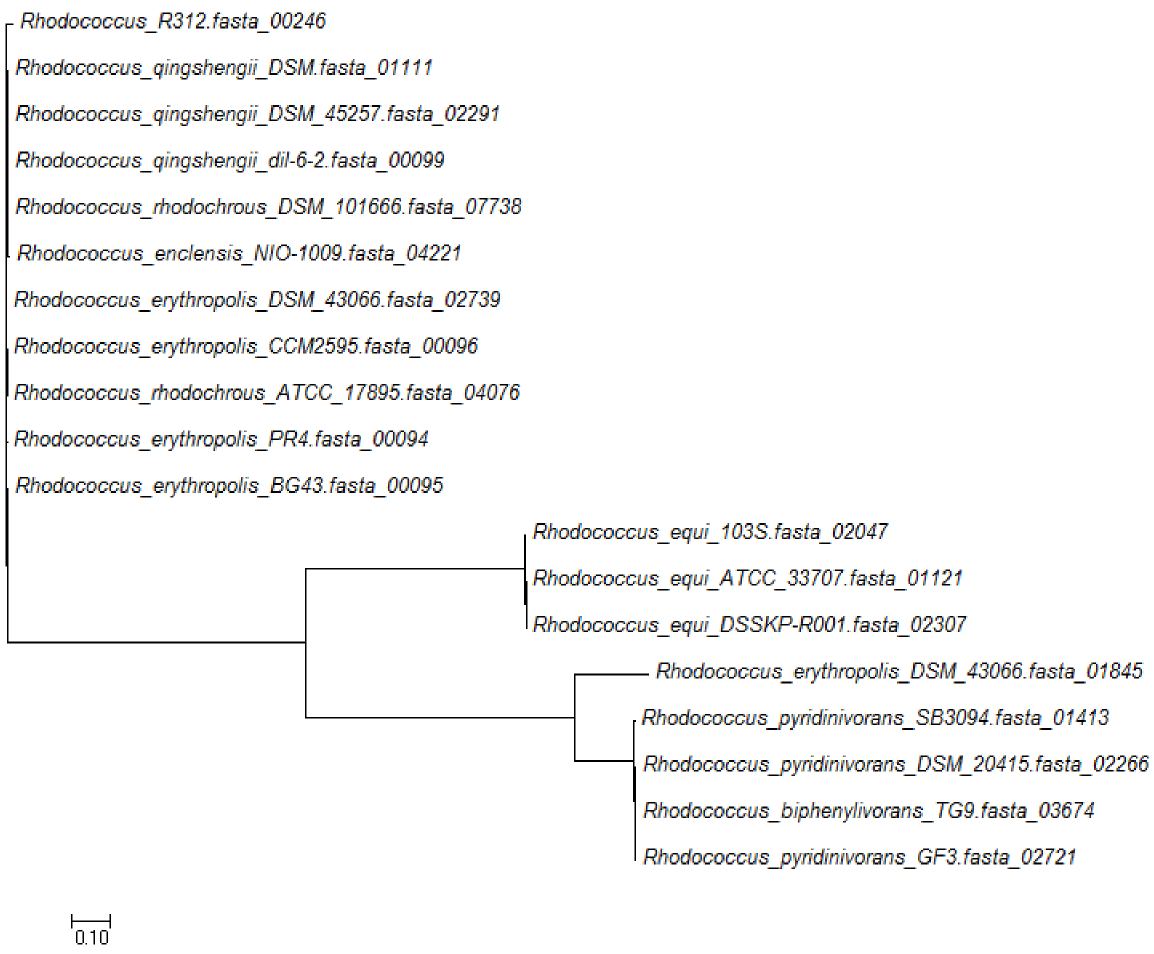

Figure S2: Phylogenetic tree of discovered oleate hydratases from *Rhodococcus* showing three clades: (i) *erythropolis*-clade, (ii) *equi*-clade, (iii) *pyridinivorans*-clade.

While the *erythropolis*- and *equi*-clades are built up uniformly, the *pyridinivorans*-clade is split in two sub-groups. Here, the second oleate hydratase from *R. erythropolis* DSM 43066 forms its own sub-group, while the oleate hydratases from *pyridinivorans* and *biphenylivorans* are closely related. Oleate hydratase protein sequences were aligned using muscle (version 3.8.31)(Edgar 2004). Distance-matrix was calculated by FastTree (version 2.1.9, JTT+CAT model) and then visualised by MEGA (Price et al. 2010; Kumar et al. 2016).

```
Rhodococcus R312                            KLSHKAYMIGAGIGNLSAAVYLIRDGKWSGEDIIIMG-LDMHGANDGESAATFQHQYGHR    62
Rhodococcus enclensis NIO-1009              KLSHKAYMIGAGIGNLSAAVYLIRDGEWSGEDITIMG-LDMHGANDGESAATFQHQYGHR    62
Rhodococcus qingshengii DSM 46766           KLSHKAYMIGAGIGNLSAAVYLIRDGEWSGEDITIMG-LDMHGANDGESAATFQHQYGHR    62
Rhodococcus qingshengii DSM 45257           KLSHKAYMIGAGIGNLSAAVYLIRDGEWSGEDITIMG-LDMHGANDGESAATFQHQYGHR    62
Rhodococcus qingshengii dil-6-2             KLSHKAYMIGAGIGNLSAAVYLIRDGEWSGEDITIMG-LDMHGANDGESAATFQHQYGHR    62
Rhodococcus rhodochrous DSM_101666          KLSHKAYMIGAGIGNLSAAVYLIRDGEWSGEDITIMG-LDMHGANDGESAATFQHQYGHR    62
Rhodococcus erythropolis CCM2595            NLSHKAYMIGAGIGNLSAAVYLIRDGEWNGEDITIMG-LDMHGANDGESAATFQHQYGHR    62
Rhodococcus rhodochrous ATCC 17895          NLSHKAYMIGAGIGNLSAAVYLIRDGEWNGEDITIMG-LDMHGANDGESAATFQHQYGHR    62
Rhodococcus erythropolis BG43               NLSHKAYMIGAGIGNLSAAVYLIRDGEWNGEDITIMG-LDMHGANDGESAATFQHQYGHR    62
Rhodococcus erythropolis DSM 43066 HFam3    NLSHKAYMIGAGIGNLSAAVYLIRDGEWNGEDITIMG-LDMHGANDGESAATFQHQYGHR    62
Rhodococcus erythropolis PR4                NLSHKAYMIGAGIGNLSAAVYLIRDGEWNGEDITIMG-LDMHGANDGESAATFQHQYGHR    62
Rhodococcus equi 103S                       PKQAHIWIVGGGIAGMAAAVFAIRDAGVPGENVHILEQLDIEGGS----------LDGAR    54
Rhodococcus equi ATCC 33707                 PKQAHIWIVGGGIAGMAAAVFAIRDAGVPGENVHILEQLDIEGGS----------LDGAR    54
Rhodococcus equi DSSKP-R001                 PKQAHIWIVGGGIAGMAAAVFAIRDAGVPGENVHILEQLDIEGGS----------LDGAR    54
Em-OAH1                                     YDNSKIYIIGSGIAGMSAAYYFIRDGHVPAKNITFLEQLHIDGGS----------LDGAG   109
Rhodococcus erythropolis DSM 43066 HFam2    VDGKTAWFVGSGLASLAGAAFMIRDGQMAGNNITVLERLKLPGGA----------LDGIK    69
Rhodococcus pyridinivorans SB3094           VENKTAWFVGAGLTSMASAVFMIRDGQLSGDKITILERLDLPGGA----------LDGIK    69
Rhodococcus pyridinivorans GF3              VENKTAWFVGAGLTSMASAVFMIRDGQLSGDKITILERLDLPGGA----------LDGIK    69
Rhodococcus biphenylivorans TG9             VENKTAWFVGAGLTSMASAVFMIRDGQLSGDKITILERLDLPGGA----------LDGIK    69
Rhodococcus pyridinivorans DSM 20415        VENKTAWFVGAGLTSMASAVFMIRDGQLSGDKITILERLDLPGGA----------LDGIK    69
                                                   :::*.*: .::.* : ***.    ...: .:  *.: *.            *
```

Figure S3: CLUSTAL multiple sequence alignment of *Rhodococcus oleate* hydratases compared to *Em*-OAH1 in the range of conserved FAD-binding region (G69-E96) (Madeira et al. 2019).

```
Rhodococcus R312                            ELGNDAGFINRGGRMLNEETYENLWDVLSAIPSLDNPG-KSVTDDILDFDHAHPTHDVAR   121
Rhodococcus enclensis NIO-1009              ELGNDAGFINRGGRMLNEETYENLWDVLSVVPSLDNPG-KSVTDDILDFDHAHPTHDVAR   121
Rhodococcus qingshengii DSM 46766           ELGNDAGFINRGGRMLNEETYENLWDVLSVVPSLDNPG-KSVTDDILDFDHAHPTHDVAR   121
Rhodococcus qingshengii DSM 45257           ELGNDAGFINRGGRMLNEETYENLWDVLSVVPSLDNPG-KSVTDDILDFDHAHPTHDVAR   121
Rhodococcus qingshengii dil-6-2             ELGNDAGFINRGGRMLNEETYENLWDVLSVVPSLDNPG-KSVTDDILDFDHAHPTHDVAR   121
Rhodococcus rhodochrous DSM 101666          ELGNDAGFINRGGRMLNEETYENLWDVLSVVPSLDNPG-KSVTDDILDFDHAHPTHDVAR   121
Rhodococcus erythropolis CCM2595            ELGNDAGFINRGGRMLNEETYENLWDVLSAVPSLDNPG-KSVTDDILDFDHAHPTHDVAR   121
Rhodococcus rhodochrous ATCC 17895          ELGNDAGFINRGGRMLNEETYENLWDVLSAVPSLDNPG-KSVTDDILDFDHAHPTHDVAR   121
Rhodococcus erythropolis BG43               ELGNDAGFINRGGRMLNEETYENLWDILSAVPSLDNPG-KSVTDDILDFDHAHPTHDVAR   121
Rhodococcus erythropolis DSM 43066 HFam3    ELGNDAGFINRGGRMLNEETYENLWDVLSAVPSLDNPG-KSVTDDILDFDHAHPTHDVAR   121
Rhodococcus erythropolis PR4                ELGNDAGFINRGGRMLNEETYENLWDILSAVPSLDNPG-KSVTDDILDFDHAHPTHDVAR   121
Rhodococcus equi 103S                       SPAVTDGWVTRGGRMLEEEAYRCTWNLFESIPSLENPD-ISVRQEIRDFNEKVRTDDKAR   113
Rhodococcus equi ATCC 33707                 SPAVTDGWVTRGGRMLEEEAYRCTWNLFESIPSLENPD-ISVRQEIRDFNEKVRTDDKAR   113
Rhodococcus equi DSSKP-R001                 SPAVTDGWVTRGGRMLEEEAYRCTWNLFESIPSLENPD-ISVRQEIRDFNEKVRTDDKAR   113
Em-OAH1                                     NP--TDGYIIRGGREMD-MTYENLWDMFQDIPALEMPAPYSVLDEYRLINDNDSNYSKAR   166
Rhodococcus erythropolis DSM 43066 HFam2    EP--EKGFVIRGGREME-DHFECLWDLFRSVPSIEVED-ASVLDEFYWLNKDDPNYSLQR   125
Rhodococcus pyridinivorans SB3094           KP--DKGFVIRGGREME-DHMECLWDLFRTIPSLEVD--GSVLDEFYWLNKDDPNYSLNR   124
Rhodococcus pyridinivorans GF3              KP--DKGFVIRGGREME-DHMECLWDLFRTIPSLEVD--GSVLDEFYWLNKDDPNYSLNR   124
Rhodococcus biphenylivorans TG9             KP--DKGFVIRGGREME-DHMGCLWDLFRTIPSLEVD--GSVLDEFYWLNKDDPNYSLNR   124
Rhodococcus pyridinivorans DSM 20415        KP--DKGFVIRGGREME-DHMECLWDLFRTIPSLEVD--GSVLDEFYWLNKDDPNYSLNR   124
                                            .     *:: **** ::       *:::  :*:::     ** ::   ::.   . .  *
```

Figure S4: CLUSTAL multiple sequence alignment of *Rhodococcus* oleate hydratases compared to *Em*-OAH1 in the area of conserved R118GGREM123 (*Em*-OAH1) motif responsible for cofactor binding and presumably involved in catalysis (Madeira et al. 2019).

```
Rhodococcus R312                            VTTSSHELINEISRITKQLPGN------ALNTFVDSNVLLSIVVHHQPHYHAQKENEGVF   410
Rhodococcus enclensis NIO-1009              VTTSSHELINEISRITKQLPGN------ALNTFVDSNVLLSIVVHHQPHYHAQKENEGVF   410
Rhodococcus qingshengii DSM 46766           VTTSSHELINEISRITKQLPGN------ALNTFVDSNVLLSIVVHHQPHYHAQKENEGVF   410
Rhodococcus qingshengii DSM 45257           VTTSSHELINEISRITKQLPGN------ALNTFVDSNVLLSIVVHHQPHYHAQKENEGVF   410
Rhodococcus qingshengii dil-6-2             VTTSSHELINEISRITKQLPGN------ALNTFVDSNVLLSIVVHHQPHYHAQKENEGVF   410
Rhodococcus rhodochrous DSM 101666          VTTSSHELINEISRITKQLPGN------ALNTFVDSNVLLSIVVHHQPHYHAQKENEGVF   410
Rhodococcus erythropolis CCM2595            VTTSSHELINEISRITKQLPGN------ALNTFVDSNVLLSIVVHHQPHYHAQKENEGVF   410
Rhodococcus rhodochrous ATCC 17895          VTTSSHELINEISRITKQLPGN------ALNTFVDSNVLLSIVVHHQPHYHAQKENEGVF   410
Rhodococcus erythropolis BG43               VTTSSHELINEISRITKQLPGN------ALNTFVDSNVLLSIVVHHQPHYHAQKENEGVF   410
Rhodococcus erythropolis DSM 43066 HFam3    VTTSSHELINEISRITKQLPGN------ALNTFVDSNVLLSIVVHHQPHYHAQKENEGVF   410
Rhodococcus erythropolis PR4                VTTSSHELINEISRITKQLPGN------ALNTFVDSNVLLSIVVHHQPHYHAQKENEGVF   410
Rhodococcus equi 103S                       LTMRGDKLLRRITEYTGNEPGT-----GALTTWFESGWHLSTVVAYQPHFPQQPEGVYTL   391
Rhodococcus equi ATCC 33707                 LTMRGDKLLRRITEYTGNEPGT-----GALTTWFESGWHLSTVVAYQPHFPQQPEGVYTL   391
Rhodococcus equi DSSKP-R001                 LTMRGDKLLRRITEYTGNEPGT-----GALTTWFESGWHLSTVVAYQPHFPQQPEGVYTL   391
Em-OAH1                                     LTCKPSALIDKLKEYSVNDPYSGKTVTGGIITITDSNWLMSFTCNRQPHFPEQPDDVLVL   453
Rhodococcus erythropolis DSM 43066 HFam2    VTTLDARIPEYIEKICKRDPFSGRVVTGGIVTARDSKWLMSWTVNRQPHFKQQPKDQIVV   408
Rhodococcus pyridinivorans SB3094           VTTIGPEIPRYIKKIAKRDPFSGNIVTGGIVTAKDSSWLLSWTVNRQPHFKAQAPDEIVV   407
Rhodococcus pyridinivorans GF3              VTTIGPEIPRYIKKIAKRDPFSGNIVTGGIVTAKDSSWLLSWTVNRQPHFKAQAPDEIVV   407
Rhodococcus biphenylivorans TG9             VTTIGPEIPRYIKKIAKRDPFSGNIVTGGIVTAKDSSWLLSWTVNRQPHFKAQAPDEIVV   407
Rhodococcus pyridinivorans DSM 20415        VTTIGPEIPRYIKKIAKRDPFSGNIVTGGIVTAKDSSWLLSWTVNRQPHFKAQAPDEIVV   407
                                            :*      :   : .   . * .      .: *  :*   :* .    ***:  *  .  ..
```

Figure S5: CLUSTAL multiple sequence alignment of *Rhodococcus* oleate hydratases compared to *Em*-OAH1 in the area of conserved amino acids involved in carboxylate binding (T436 and N438) (Madeira et al. 2019).

Table S3: Molecular structures of tested fatty acids.

#	Name	molecular structure
1	myristoleic acid<br>14:1, *cis*-9	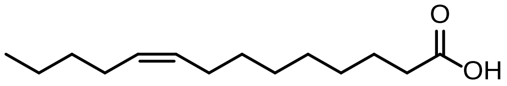
2	palmitoleic acid<br>16:1, *cis*-9	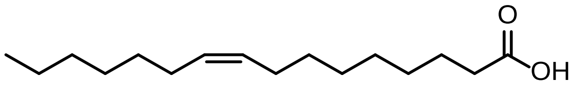
3	oleic acid<br>18:1, *cis*-9	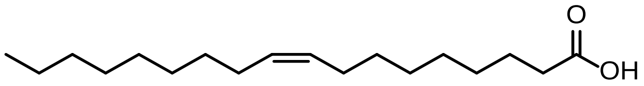
4	linoleic acid<br>18:2, *cis*-9,12	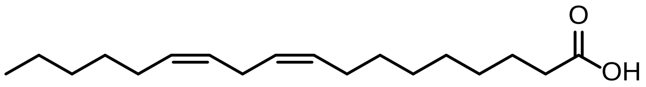
5	pinolenic acid<br>18:3, *cis*-5,9,12	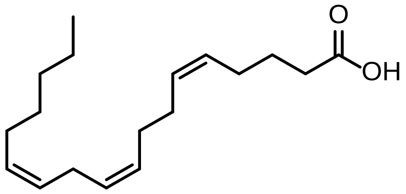
6	*cis*-vaccenic acid<br>18:1, *cis*-11	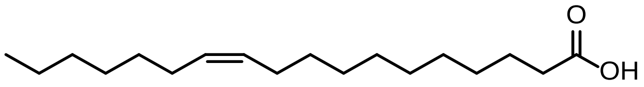
7	*trans*-vaccenic acid<br>18:1, *trans*-11	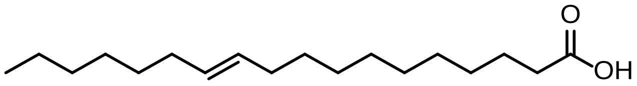
8	γ-linolenic acid<br>18:3, *cis*-6,9,12	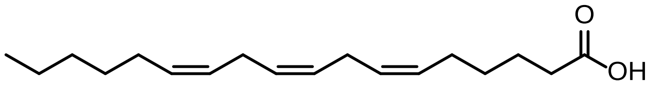
9	ricinoleic acid<br>18:1, *cis*-9, (*R*)-12-OH	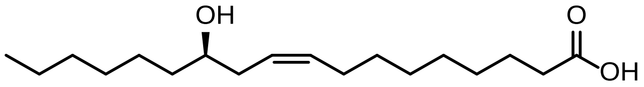
10	*cis*-11-eicosenoic acid<br>20:1, *cis*-11	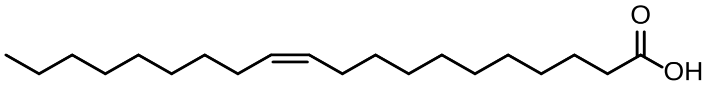
11	*cis*-11,14-eicosadienoic acid<br>20:2, *cis*-8,11	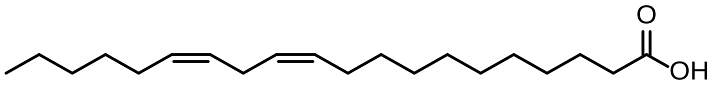
12	*cis*-8,11,14-eicosatrienoic acid<br>20:3, *cis*-8,11,14	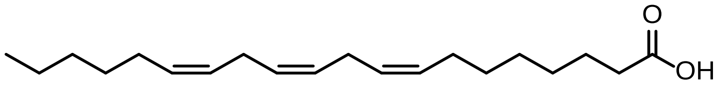
13	arachidonic acid<br>20:4, *cis*-5,8,11,14	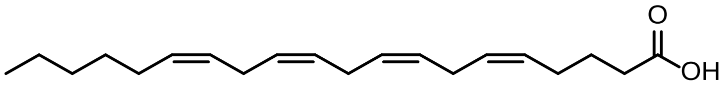
14	erucic acid<br>22:1, *cis*-13	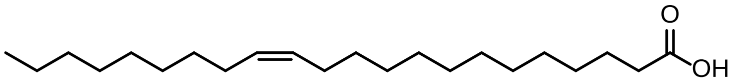
15	nervonic acid<br>24:1, *cis*-15	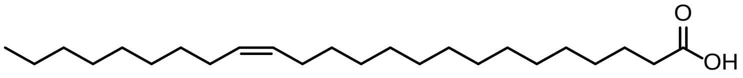

GC-MS data of hydroxylated fatty acids:

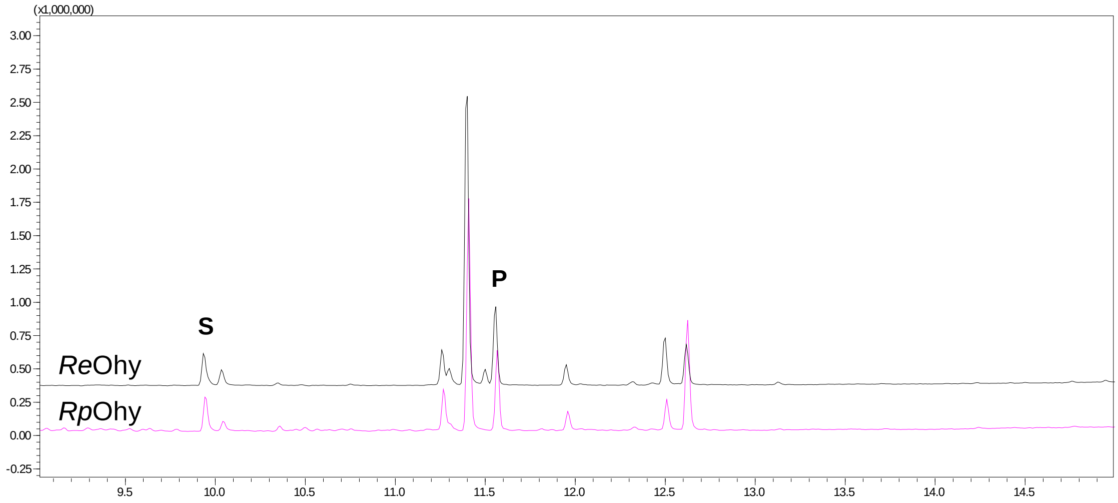

Figure S6: GC-MS chromatograms showing the products of *E. coli* whole-cell bioconversions of *Re*Ohy (black) and *Rp*Ohy (pink) of fatty acid #1 – myrestoleic acid. S: substrate, P: product.

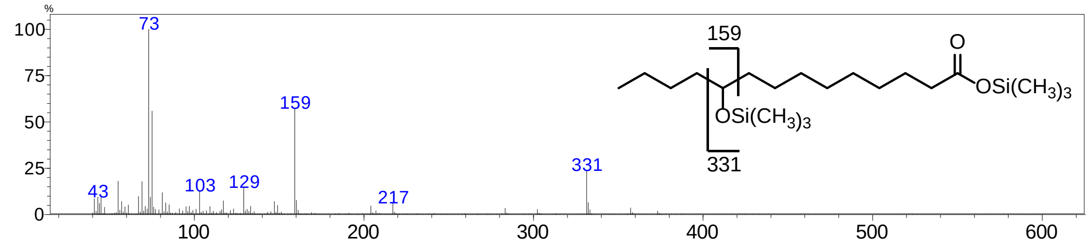

Figure S7: Mass spectrum of silylated, hydroxylated myrestoleic acid with significant fragment ions: *m/z* 159 and 331.

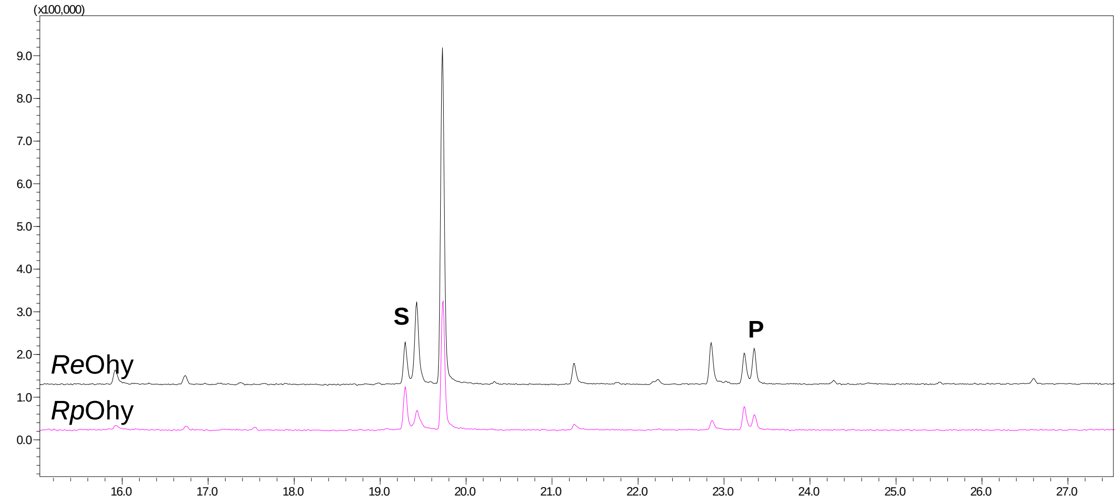

Figure S8: GC-MS chromatograms showing the products of *E. coli* whole-cell bioconversions of *Re*Ohy (black) and *Rp*Ohy (pink) of fatty acid #2 – palmitoleic acid. S: substrate, P: product.

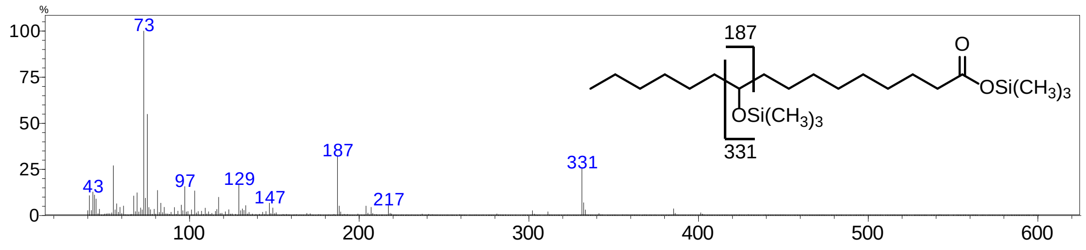

Figure S9: Mass spectrum of silylated, hydroxylated palmitoleic acid with significant fragment ions: *m/z* 187 and 331.

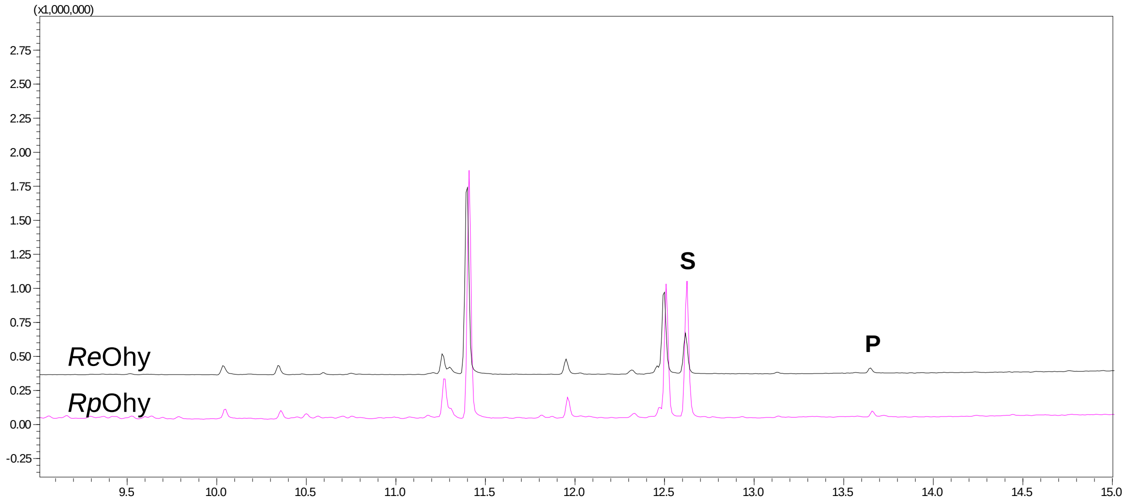

Figure S10: GC-MS chromatograms showing the products of *E. coli* whole-cell bioconversions of *Re*Ohy (black) and *Rp*Ohy (pink) of fatty acid #3 – oleic acid. S: substrate, P: product.

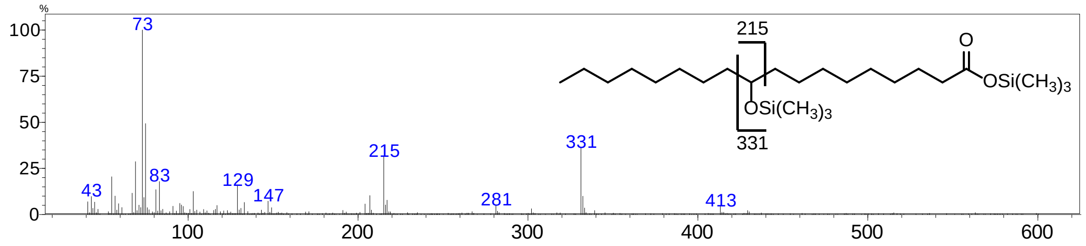

Figure S11: Mass spectrum of silylated, hydroxylated oleic acid with significant fragment ions: *m/z* 215 and 331.

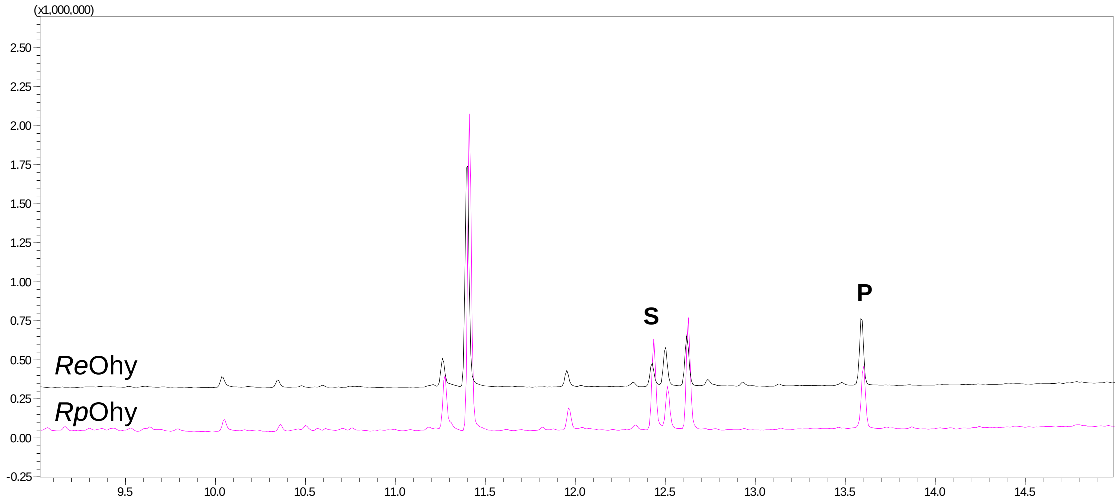

Figure S12: GC-MS chromatograms showing the products of *E. coli* whole-cell bioconversions of *Re*Ohy (black) and *Rp*Ohy (pink) of fatty acid #4 – linoleic acid. S: substrate, P: product.

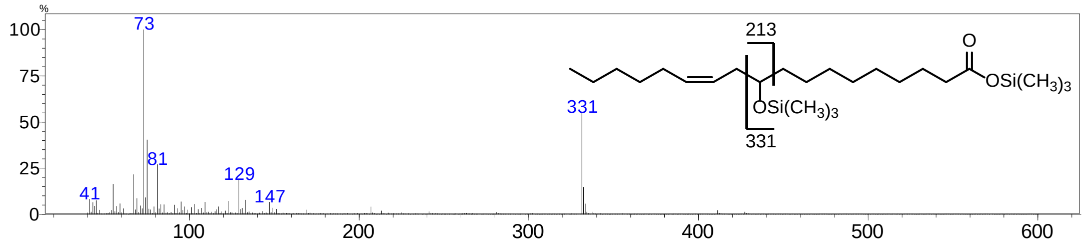

Figure S13: Mass spectrum of silylated, hydroxylated linoleic acid with significant fragment ion: *m/z* 331. Ion 213 was not detected due to an re-arrangement following the allyl-position of the alcohol.

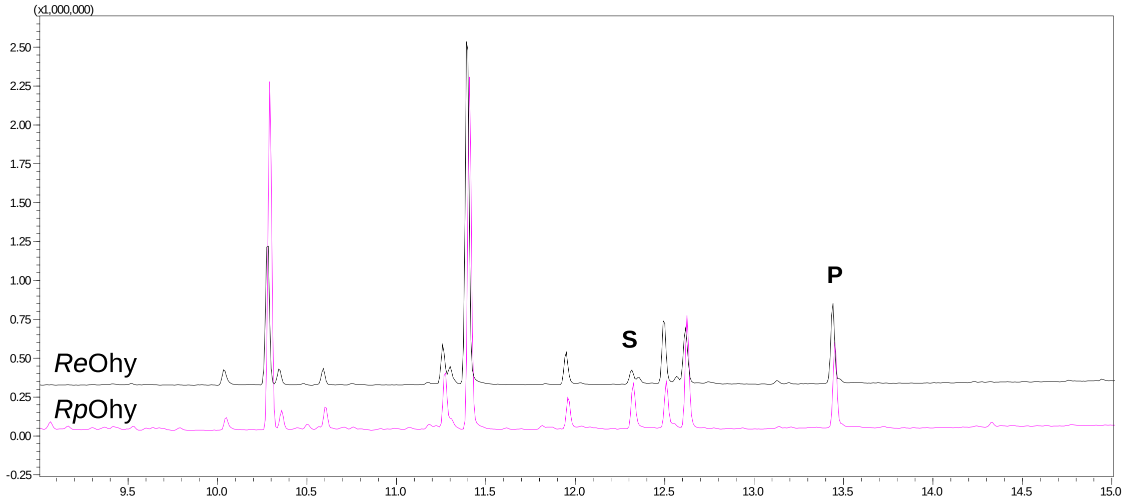

Figure S14: GC-MS chromatograms showing the products of *E. coli* whole-cell bioconversions of *Re*Ohy (black) and *Rp*Ohy (pink) of fatty acid #5 – pinolenic acid. S: substrate, P: product.

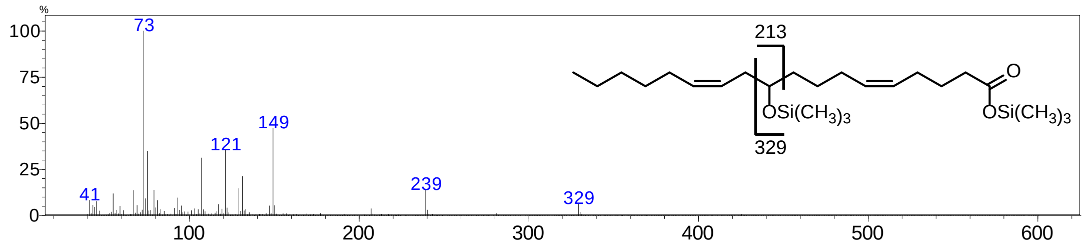

Figure S15: Mass spectrum of silylated, hydroxylated pinolenic acid with significant fragment ions highlighted: *m/z* 329. Ion 213 was not detected due to an re-arrangement following the allyl-position of the alcohol.

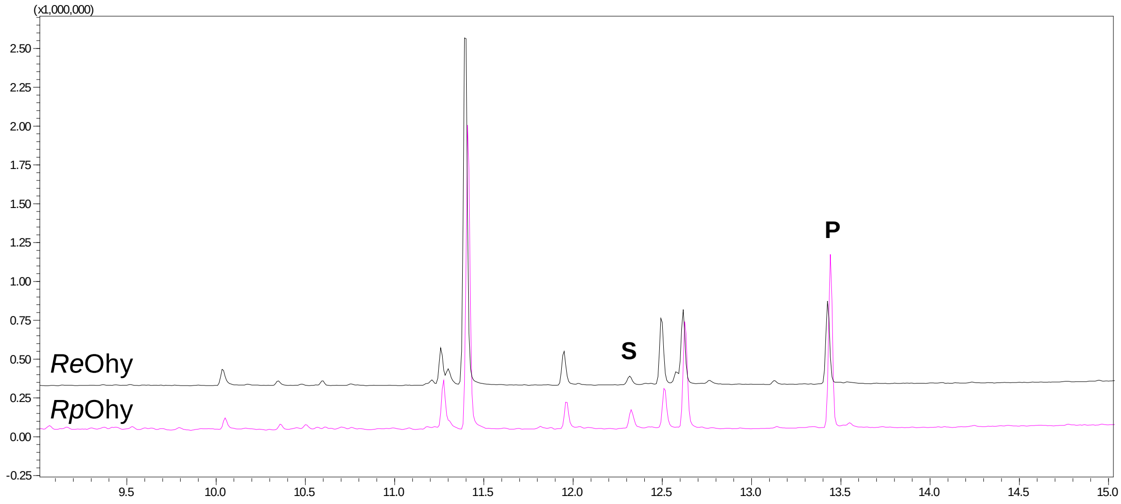

Figure S16: GC-MS chromatograms showing the products of *E. coli* whole-cell bioconversions of *Re*Ohy (black) and *Rp*Ohy (pink) of fatty acid #8 – γ-linolenic acid. S: substrate, P: product.

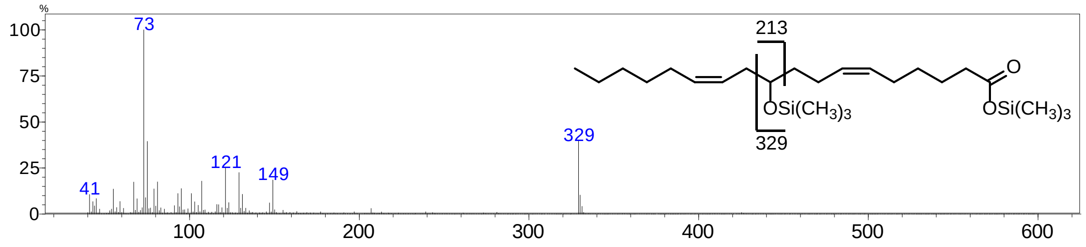

Figure S17: Mass spectrum of silylated, hydroxylated γ-linolenic acid with significant fragment ions highlighted: *m/z* 329. Ion 213 was not detected due to an re-arrangement following the allyl-position of the alcohol.

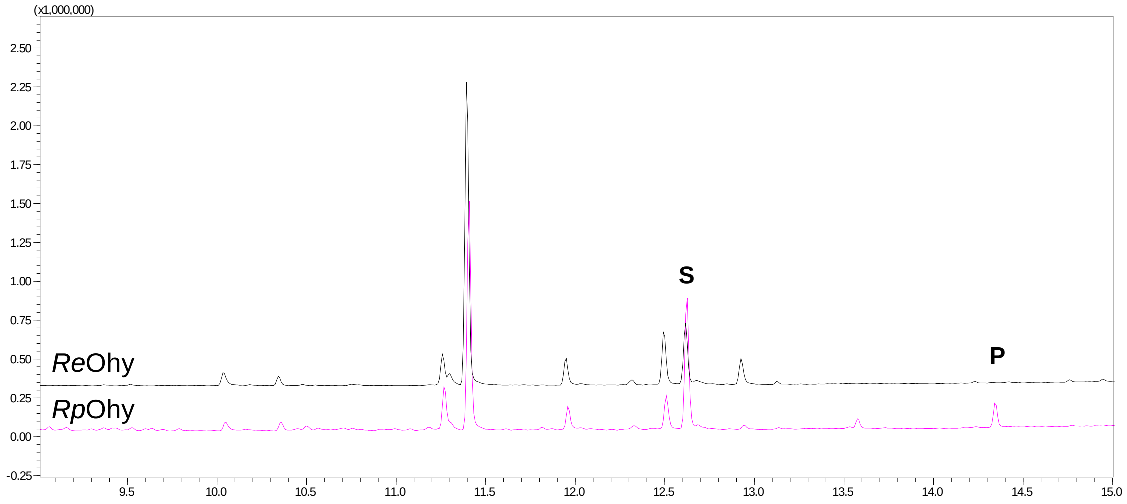

Figure S18: GC-MS chromatograms showing the products of *E. coli* whole-cell bioconversions of *Re*Ohy (black) and *Rp*Ohy (pink) of fatty acid #9 – ricinoleic acid. S: substrate, P: product.

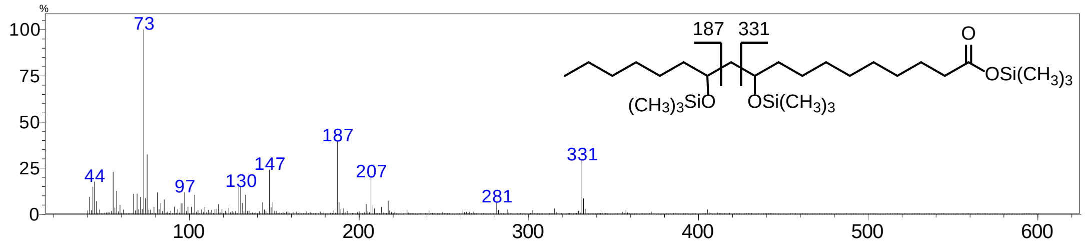

Figure S19: Mass spectrum of silylated, hydroxylated ricinoleic acid with significant fragment ions highlighted: *m/z* 187 and 331.

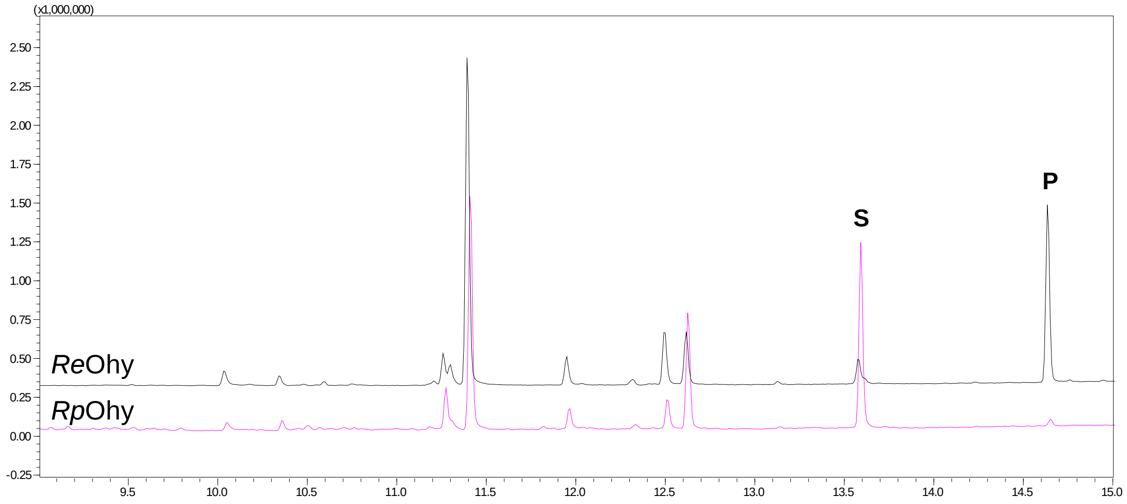

Figure S20: GC-MS chromatograms showing the products of *E. coli* whole-cell bioconversions of *Re*Ohy (black) and *Rp*Ohy (pink) of fatty acid #10 – *cis*-11-eicosenoic acid. S: substrate, P: product.

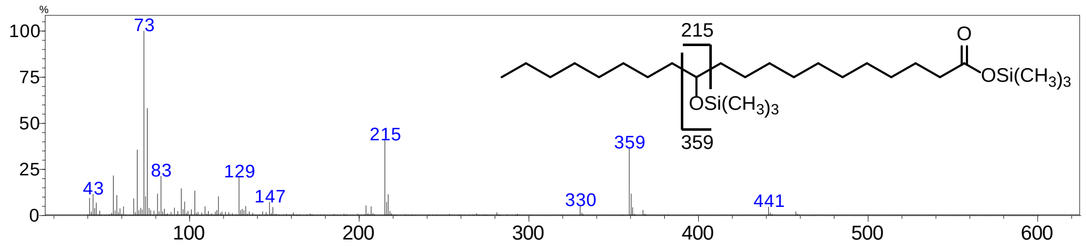

Figure S21: Mass spectrum of silylated, hydroxylated *cis*-11-eicosenoic acid with significant fragment ions highlighted: *m/z* 215 and 359.

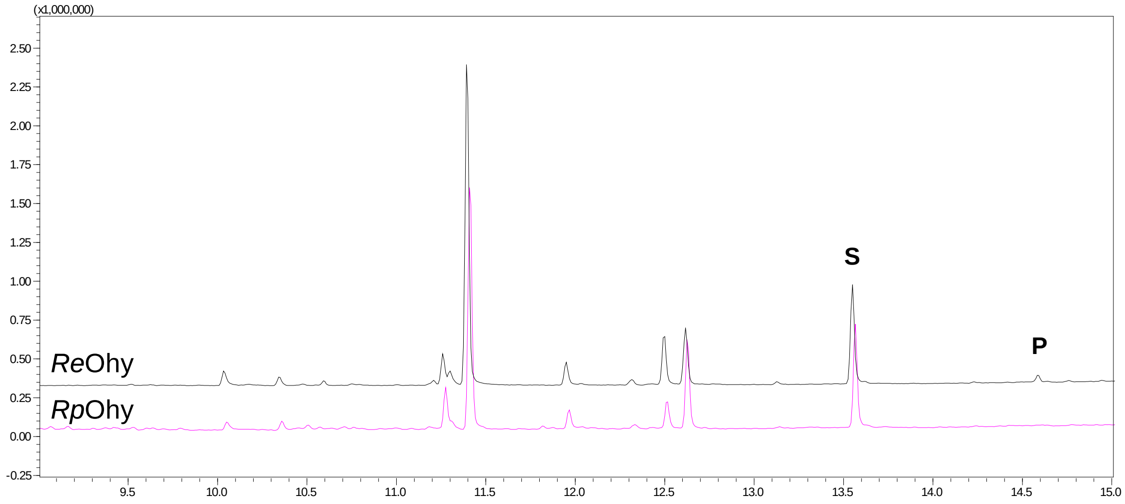

Figure S22: GC-MS chromatograms showing the products of *E. coli* whole-cell bioconversions of *Re*Ohy (black) and *Rp*Ohy (pink) of fatty acid #11 – *cis*-11,14-eicosadienoic acid. S: substrate, P: product.

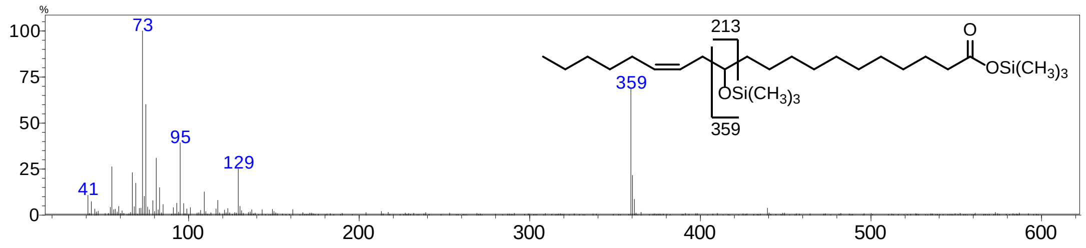

Figure S23: Mass spectrum of silylated, hydroxylated *cis*-11,14-eicosadienoic acid with significant fragment ions highlighted: *m/z* 359. Ion 213 was not detected due to a re-arrangement following the allyl-position of the alcohol.

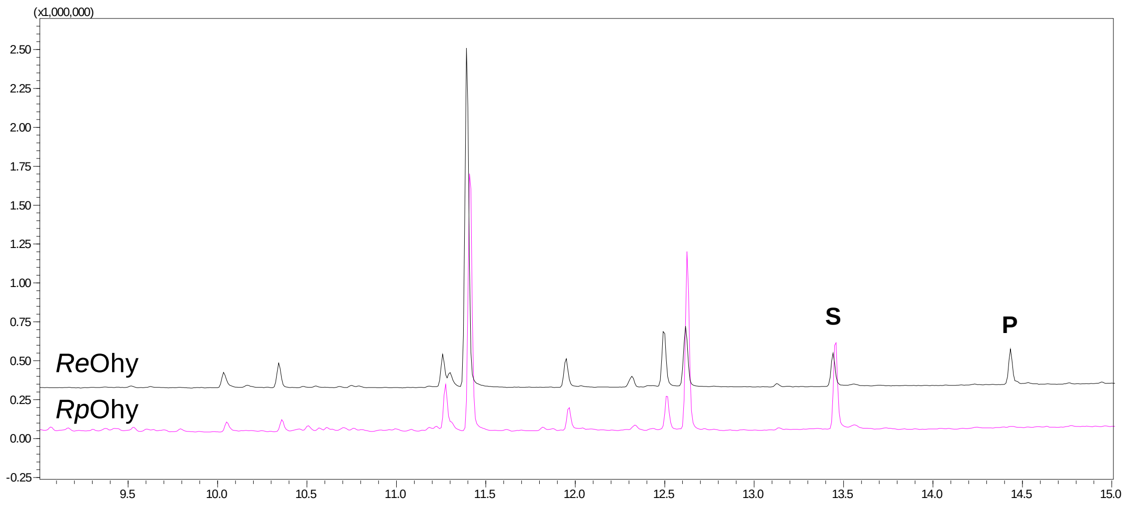

Figure S24: GC-MS chromatograms showing the products of *E. coli* whole-cell bioconversions of *Re*Ohy (black) and *Rp*Ohy (pink) of fatty acid #12 – *cis*-8,11,14-eicosatrienoic acid. S: substrate, P: product.

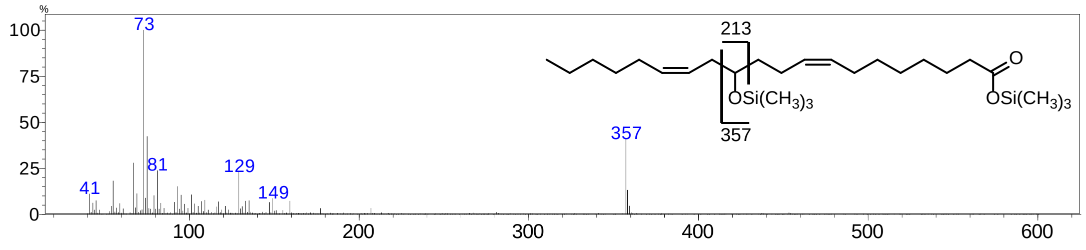

Figure S25: Mass spectrum of silylated, hydroxylated *cis*-11,14-eicosadienoic acid with significant fragment ions highlighted: *m/z* 357. Ion 213 was not detected due to a re-arrangement following the allyl-position of the alcohol.

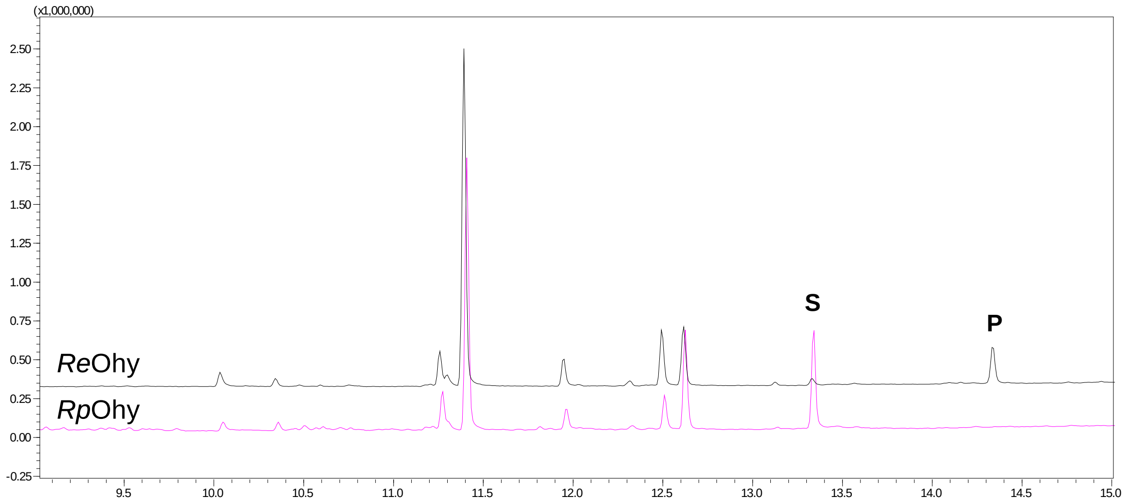

Figure S26: GC-MS chromatograms showing the products of *E. coli* whole-cell bioconversions of *Re*Ohy (black) and *Rp*Ohy (pink) of fatty acid #13 – arachidonic acid. S: substrate, P: product.

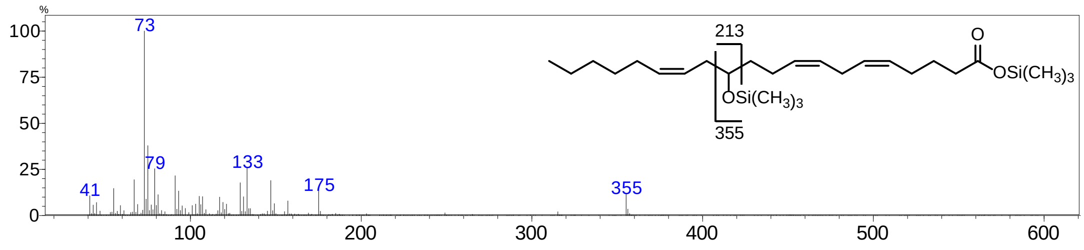

Figure S27: Mass spectrum of silylated, hydroxylated arachidonic acid with significant fragment ions highlighted: *m/z* 355. Ion 213 was not detected due to a re-arrangement following the allyl-position of the alcohol.

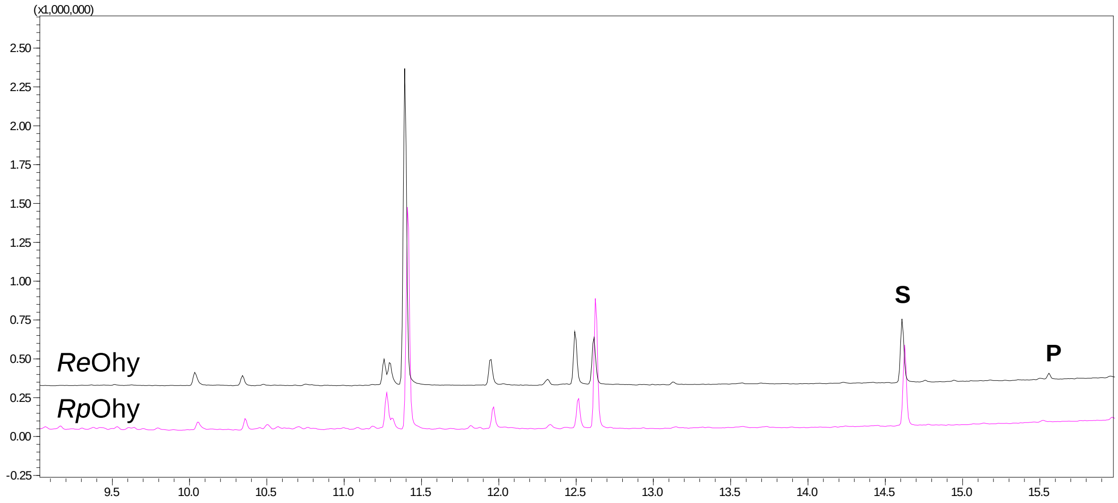

Figure S28: GC-MS chromatograms showing the products of *E. coli* whole-cell bioconversions of *Re*Ohy (black) and *Rp*Ohy (pink) of fatty acid #14 – erucic acid. S: substrate, P: product.

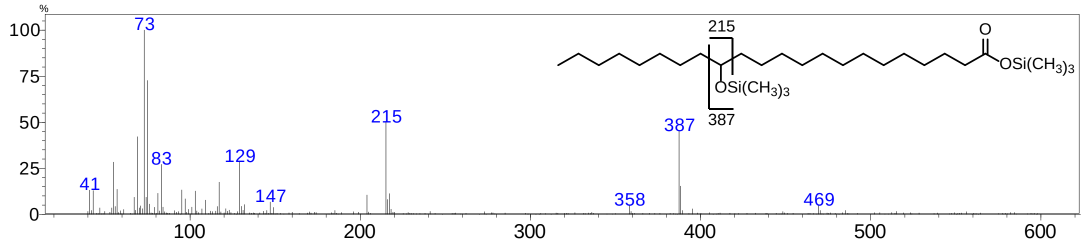

Figure S29: Mass spectrum of silylated, hydroxylated erucic acid with significant fragment ions highlighted: *m/z* 215 and 387.

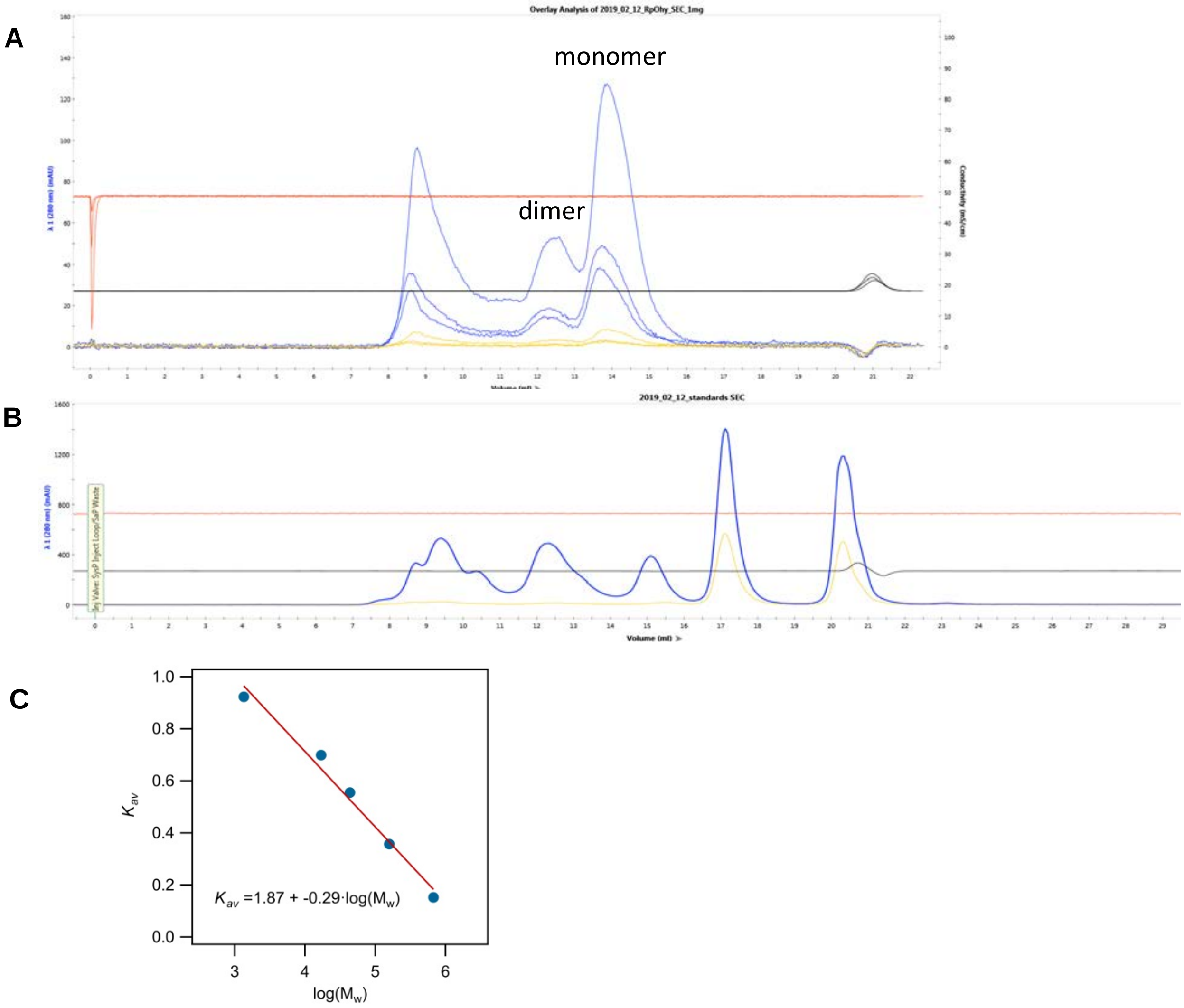

Figure S30: Size exclusion chromatography of *Rp*Ohy
A) Analytical SEC of 1, 5 and 10 mg *Rp*Ohy; B) Protein standards SEC with Thyroglobulin (670 kDa), γ-globulin (158 kDa), Ovalbumin (44 kDa), Myoglobin (17 kDa) and Vitamin B12 (1.35 kDa); C) SEC calibration curve. SEC conditions: Superdex S200 10/300 GL running with 0.5 ml/min 20 mM Tris pH 8.0 containing 250 mM NaCl.

Table S4: Molecular weight estimation *Rp*Ohy

Peak	V_e (ml)	K_{av}	Mw calculated (kDa)	Apparent oligomeric state
1	8.76	0.110	1,200	18
2	12.4	0.369	153	2.3
3	13.9	0.473	67.4	0.99

V_e is the elution volume. Void volume V_o = 7.2 ml, total column volume V_t = 21.4 ml. Gel-phase distribution coefficient $K_{av} = \frac{V_e - V_o}{V_t - V_o}$

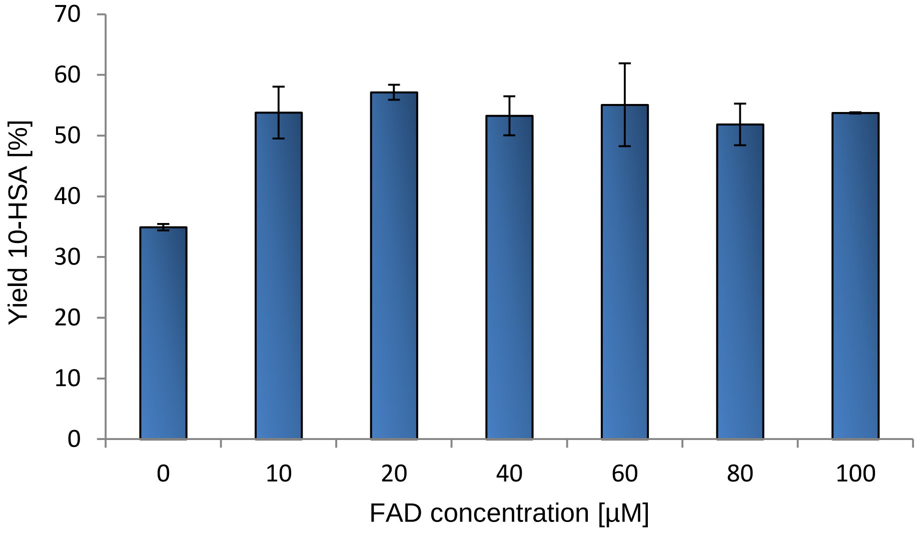

Figure S31: Influence of FAD addition to biotransformation of oleic acid to 10-hydroxystearic acid. *Reaction conditions*: 5 µM *Rp*Ohy, 20 mM acetate buffer (pH 5), 500 µM oleic acid, 1% DMSO, 5 h, 30 °C.

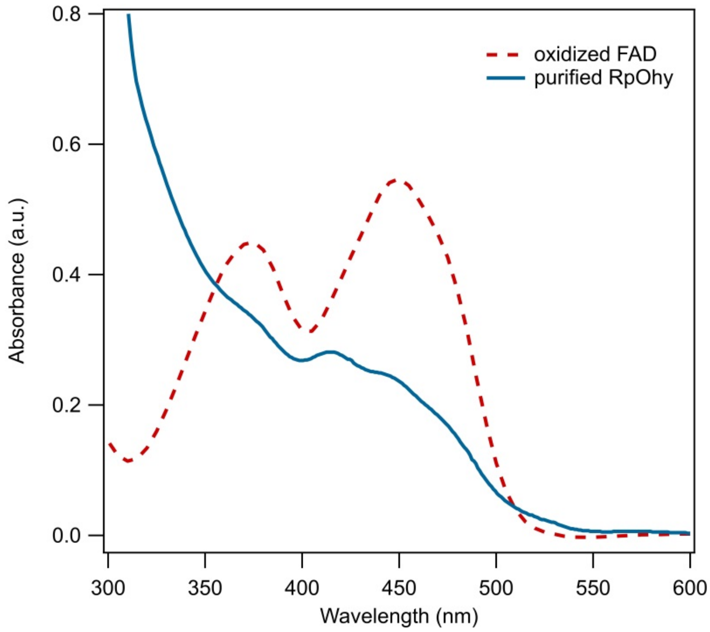

Figure S32: UV-visible absorption spectrum of purified *Rp*Ohy in the range from 300 – 600 nm (blue, solid line) with oxidised FAD as a reference (red, dashed line).

Nucleotide sequence *Rp*Ohy

```
ATGTACTACAGCAGTGGAAACTACGAAGCCTTCGCCCGCCCCCGCAAGCCCGAGGGAGTCGAGAACAAGACCGCCTGGTTCGTCGGCG
CCGGGCTCACCTCGATGGCCTCGGCGGTGTTCATGATCCGCGACGGGCAACTCTCCGGCGACAAGATCACAATCCTCGAGCGCCTGGA
CCTGCCCGGCGGCGCGCTGGACGGTATCAAGAAGCCGGACAAAGGGTTTGTCATCCGCGGCGGGCGCGAGATGGAAGACCACATGGAG
TGCCTCTGGGATCTGTTCCGCACCATCCCGTCGCTCGAGGTCGACGGATCCGTCCTCGACGAGTTCTACTGGCTCAACAAAGACGACC
CCAACTACTCACTCAACCGGGTCACACACCGGCAGGGCGAGGAGTTCGTCACCAACAATGAGTTCGGCCTGAGCGAGAAAGCTCAGAA
GGAGTTGGTCAAGGTCTTTCTCGCCTCGCGTGAGGAGATGGAAGACAAGCGCATCGACGAGATCTTCGGTGAAGAGTTCCTGAGCAGC
AACTTCTGGCTCTACTGGCGGACGATGTTCGCGTTCGAGAACTGGCACTCCGCACTCGAGCTGAAGCTCTACCTGCACCGGTTCGTGC
ACCACATCGGCGGGCTCCCCGACCTTTCGGCGCTGAAGTTCACAAAGTACAACCAGTACGAATCGCTCGTGCTGCCCATGTACCGCTG
GCTCCTGGACCAGGGCGTGAGATTCGAGTTCTCCACCGAGGTCACCGACATCGACTTCGTTTTCGACGGCGACCGTAAACAGGCCACC
CGCATCCACTGGACAAAGGGCGGCGTGCCCGGCGGCGTCGACCTCGGTCCCGACGACCTCGTGCTCGCCACGATCGGTTCGCTCACAG
AGAACTCCGACGACGGCACCCACCACAATGCGGCCAGACTCGACGAAGGTCCCGCGCCGGCGTGGGACCTGTGGCGGCGCATCGCGGC
CAAGCACCCCTCTTTCGGACGCCCCGAGGTGTTCTGCGGCGACATCTCGAAGACGAAGTGGGAGTCAGCGACGGTCACCACCATCGGC
CCTGAGATCCCGAGGTACATCAAGAAGATCGCCAAGCGCGACCCGTTCTCCGGCAACATCGTCACCGGCGGCATCGTCACCGCGAAAG
ACTCGTCCTGGCTGCTCAGCTGGACAGTCAACCGCCAGCCGCACTTCAAAGCGCAAGCACCCGACGAGATCGTCGTGTGGGTCTACGG
CCTGTTCGTAGACGTGCCCGGCGACTTCACCGGCAAGACAATGCAGGAGTCGACCGGCGAAGAGATCACCCAAGAATGGCTCTATCAC
TTGGGTGTGCCCGTCGAGGACATCCCGGAGCTCGCGGCAAACGGCGCGAAGACCGTGCCTGTGATGATGCCCTACGTCACCTCGTTCT
TCATGCCCCGCACCGCCGGTGACCGCCCCGACGTGGTGCCCGAGGGCGCAGTGAACTTCGCGTTCATCGGCCAGTTCGCCGAAACCAC
GCGCGACACGATCTTCACCACCGAGTACTCGGTGCGTACTGCGATGGAGGCCGCCTACCAGCTGCTGGGCATCGATCGCGGCGTGCCG
GAGGTGTTCAACTCCACCTATGATCTCAGGTTCCTGCTCGAGGCCACCGCGCGCTTGCGCGACGGCGAGGAGGTAGAGCTCCCCGGCC
CGAAGTTCGTCGGCAACCGCATCATCAAGCACCTCGACCACACTCAGATCGGACAACTCCTCACCGACTTCGGAGTCATCCCCGAGCT
CGACGGCACGACGAAACGAGTCCGTGCCGATGACGCCGGAGCCTGA
```

Amino-acid sequence *Rp*Ohy

```
MYYSSGNYEAFARPRKPEGVENKTAWFVGAGLTSMASAVFMIRDGQLSGDKITILERLDLPGGALDGIKKPDKGFVIRGGREMEDHME
CLWDLFRTIPSLEVDGSVLDEFYWLNKDDPNYSLNRVTHRQGEEFVTNNEFGLSEKAQKELVKVFLASREEMEDKRIDEIFGEEFLSS
NFWLYWRTMFAFENWHSALELKLYLHRFVHHIGGLPDLSALKFTKYNQYESLVLPMYRWLLDQGVRFEFSTEVTDIDFVFDGDRKQAT
RIHWTKGGVPGGVDLGPDDLVLATIGSLTENSDDGTHHNAARLDEGPAPAWDLWRRIAAKHPSFGRPEVFCGDISKTKWESATVTTIG
PEIPRYIKKIAKRDPFSGNIVTGGIVTAKDSSWLLSWTVNRQPHFKAQAPDEIVVWVYGLFVDVPGDFTGKTMQESTGEEITQEWLYH
LGVPVEDIPELAANGAKTVPVMMPYVTSFFMPRTAGDRPDVVPEGAVNFAFIGQFAETTRDTIFTTEYSVRTAMEAAYQLLGIDRGVP
EVFNSTYDLRFLLEATARLRDGEEVELPGPKFVGNRIIKHLDHTQIGQLLTDFGVIPELDGTTKRVRADDAGA
```

Nucleotide sequence synthetic *Rp*Ohy construct codon optimized for *E. coli* including his-tag

ATGGGGGGTTCTCATCATCATCATCATCATGGTATGGCTAGCATGACTGGTGGACAGCAAATGGGTCGGGATCTGTACGACGATGACG
ATAAGGATCGATGGGGATCCGAGCTCGAGATCTGCAGCTACTATTCTTCGGGCAATTATGAGGCGTTCGCGCGTCCTCGCAAACCCGA
GGGCGTAGAGAACAAGACGGCCTGGTTTGTGGGTGCAGGCTTGACGTCGATGGCCAGTGCGGTGTTCATGATCCGCGATGGACAACTG
AGTGGCGACAAAATTACTATTTTGGAACGTCTGGATCTTCCCGGAGGGGCCCTGGATGGAATTAAAAAGCCTGACAAGGGATTTGTAA
TCCGTGGAGGTCGCGAGATGGAAGATCACATGGAATGCCTTTGGGACTTGTTTCGTACCATCCCGTCCTTAGAAGTCGATGGTTCAGT
CTTGGATGAATTCTATTGGTTAAACAAGGACGACCCTAACTATTCGTTAAATCGCGTGACACACCGCCAAGGCGAAGAGTTCGTTACC
AATAATGAGTTTGGACTTTCCGAAAAGGCACAAAAAGAGTTGGTCAAAGTTTTCTTGGCGTCTCGTGAAGAAATGGAGGATAAACGTA
TCGACGAGATTTTCGGGGAAGAATTTTTATCATCAAACTTTTGGCTGTACTGGCGTACCATGTTCGCTTTTGAAAACTGGCATAGTGC
ACTGGAGTTGAAACTGTACCTGCATCGTTTCGTGCATCACATCGGCGGATTGCCTGATCTTTCCGCCTTGAAATTTACAAAGTACAAT
CAGTACGAAAGTCTTGTGCTTCCAATGTATCGTTGGTTGTTGGATCAAGGAGTACGCTTTGAGTTCTCTACCGAGGTCACAGATATCG
ATTTTGTGTTCGATGGTGACCGCAAGCAGGCCACGCGTATCCATTGGACAAAAGGAGGTGTGCCAGGCGGAGTTGATTTAGGCCCCGA
TGATTTAGTCCTGGCAACAATCGGTAGCCTGACCGAGAATTCTGACGACGGAACACACCATAATGCCGCGCGTTTAGACGAAGGACCG
GCGCCCGCTTGGGACCTGTGGCGTCGTATTGCCGCGAAACATCCGAGCTTCGGACGCCCCGAGGTTTTCTGTGGAGATATCTCAAAAA
CAAAGTGGGAATCGGCTACCGTTACGACGATCGGACCCGAAATCCCGCGCTATATTAAAAAGATCGCGAAGCGTGATCCCTTTTCGGG
AAACATTGTCACTGGGGGTATCGTGACGGCGAAAGACAGCAGCTGGTTACTTTCATGGACGGTCAACCGTCAACCACATTTCAAGGCA
CAAGCGCCAGATGAGATTGTTGTGTGGGTGTATGGTCTGTTCGTAGATGTACCAGGGGATTTTACTGGTAAGACAATGCAGGAGAGTA
CCGGGGAAGAGATCACTCAGGAGTGGTTATACCATCTGGGAGTGCCTGTAGAAGATATCCCCGAGTTAGCGGCAAACGGCGCAAAAAC
GGTACCCGTCATGATGCCTTACGTCACGTCATTCTTTATGCCTCGTACAGCTGGGGATCGCCCCGACGTGGTGCCAGAAGGTGCCGTT
AACTTTGCCTTCATTGGTCAGTTCGCTGAGACAACGCGCGATACTATCTTCACGACAGAATACTCAGTGCGTACAGCCATGGAGGCAG
CTTACCAATTGCTGGGCATCGATCGTGGCGTGCCCGAAGTGTTTAACTCTACTTATGATTTACGCTTCTTGCTGGAGGCTACCGCTCG
TTTGCGCGATGGCGAAGAGGTGGAGCTGCCCGGGCCTAAATTTGTAGGCAACCGCATCATTAAACACCTTGATCATACACAAATTGGT
CAATTATTGACTGACTTTGGCGTCATTCCGGAACTGGACGGTACTACTAAACGTGTCCGTGCTGATGACGCCGGAGCATGA

Amino-acid sequence synthetic *Rp*Ohy contstruct including His-tag

MGGSHHHHHHGMASMTGGQQMGRDLYDDDDKDRWGSELEICSYYSSGNYEAFARPRKPEGVENKTAWFVGAGLTSMASAVFMIRDGQL
SGDKITILERLDLPGGALDGIKKPDKGFVIRGGREMEDHMECLWDLFRTIPSLEVDGSVLDEFYWLNKDDPNYSLNRVTHRQGEEFVT
NNEFGLSEKAQKELVKVFLASREEMEDKRIDEIFGEEFLSSNFWLYWRTMFAFENWHSALELKLYLHRFVHHIGGLPDLSALKFTKYN
QYESLVLPMYRWLLDQGVRFEFSTEVTDIDFVFDGDRKQATRIHWTKGGVPGGVDLGPDDLVLATIGSLTENSDDGTHHNAARLDEGP
APAWDLWRRIAAKHPSFGRPEVFCGDISKTKWESATVTTIGPEIPRYIKKIAKRDPFSGNIVTGGIVTAKDSSWLLSWTVNRQPHFKA
QAPDEIVVWVYGLFVDVPGDFTGKTMQESTGEEITQEWLYHLGVPVEDIPELAANGAKTVPVMMPYVTSFFMPRTAGDRPDVVPEGAV
NFAFIGQFAETTRDTIFTTEYSVRTAMEAAYQLLGIDRGVPEVFNSTYDLRFLLEATARLRDGEEVELPGPKFVGNRIIKHLDHTQIG
QLLTDFGVIPELDGTTKRVRADDAGA

References

Aziz RK, Bartels D, Best A, DeJongh M, Disz T, Edwards RA, Formsma K, Gerdes S, Glass EM, Kubal M, Meyer F, Olsen GJ, Olson R, Osterman AL, Overbeek RA, McNeil LK, Paarmann D, Paczian T, Parrello B, Pusch GD, Reich C, Stevens R, Vassieva O, Vonstein V, Wilke A, Zagnitko O (2008) The RAST Server: Rapid annotations using subsystems technology. BMC Genomics 9. https://doi.org/10.1186/1471-2164-9-75

Edgar RC (2004) MUSCLE: multiple sequence alignment with high accuracy and high throughput. Nucleic Acids Res 32:1792–1797. https://doi.org/10.1093/nar/gkh340

Kumar S, Stecher G, Tamura K (2016) MEGA7: Molecular Evolutionary Genetics Analysis Version 7.0 for Bigger Datasets. Mol Biol Evol 33:1870–4. https://doi.org/10.1093/molbev/msw054

Madeira F, Park Y mi, Lee J, Buso N, Gur T, Madhusoodanan N, Basutkar P, Tivey ARN, Potter SC, Finn RD, Lopez R (2019) The EMBL-EBI search and sequence analysis tools APIs in 2019. Nucleic Acids Res 47:W636–W641. https://doi.org/10.1093/nar/gkz268

Price MN, Dehal PS, Arkin AP (2010) FastTree 2 - Approximately maximum-likelihood trees for large alignments. PLoS One 5:e9490. https://doi.org/10.1371/journal.pone.0009490

Schmid J, Steiner L, Fademrecht S, Pleiss J, Otte KB, Hauer B (2016) Biocatalytic study of novel oleate hydratases. J Mol Catal B Enzym 133:S243–S249. https://doi.org/10.1016/j.molcatb.2017.01.010
